# Circulant embedding with QMC: analysis for elliptic PDE with lognormal coefficients

**DOI:** 10.1007/s00211-018-0968-0

**Published:** 2018-05-03

**Authors:** Ivan G. Graham, Frances Y. Kuo, Dirk Nuyens, Rob Scheichl, Ian H. Sloan

**Affiliations:** 1 Department of Mathematical Sciences, University of Bath, Bath, BA2 7AY UK; 2 School of Mathematics and Statistics, UNSW Sydney, Sydney, NSW 2052 Australia; 3 Department of Computer Science, KU Leuven, Celestijnenlaan 200A, 3001, Leuven, Belgium

**Keywords:** 60G10, 60G60, 65C05, 65C60, 35Q86, 65D32

## Abstract

In a previous paper (Graham et al. in J Comput Phys 230:3668–3694, 2011), the authors proposed a new practical method for computing expected values of functionals of solutions for certain classes of elliptic partial differential equations with random coefficients. This method was based on combining quasi-Monte Carlo (QMC) methods for computing the expected values with circulant embedding methods for sampling the random field on a regular grid. It was found capable of handling fluid flow problems in random heterogeneous media with high stochastic dimension, but no convergence theory was provided. This paper provides a convergence analysis for the method in the case when the QMC method is a specially designed randomly shifted lattice rule. The convergence result depends on the eigenvalues of the underlying nested block circulant matrix and can be independent of the number of stochastic variables under certain assumptions. In fact the QMC analysis applies to general factorisations of the covariance matrix to sample the random field. The error analysis for the underlying fully discrete finite element method allows for locally refined meshes (via interpolation from a regular sampling grid of the random field). Numerical results on a non-regular domain with corner singularities in two spatial dimensions and on a regular domain in three spatial dimensions are included.

## Introduction

In the paper [12], the present authors proposed a new practical algorithm for solving a class of elliptic partial differential equations with coefficients given by statistically homogeneous lognormal random fields—and in particular for computing expected values of spatial functionals of such solutions. In this algorithm, the required expected value is written as a multidimensional integral of (possibly) high dimension, which is then approximated by a quasi-Monte Carlo (QMC) method. Each evaluation of the integrand is obtained by using a fully discrete finite element (FE) method to approximate the PDE. A key original feature of the method in [12] was the procedure for sampling the random field: instead of sampling the continuous random field by a truncated Karhunen–Loève (KL) expansion, the field was sampled discretely on a regular grid covering the domain and then interpolated at the (irregularly spaced) quadrature points. This completely eliminated the problem of truncation error from the KL expansion, but requires the factorisation of a dense matrix of dimension equal to the number of sample points. In [12] this was done using a circulant embedding technique. The method was found to be effective even for problems with high stochastic dimension, but [12] did not contain a convergence analysis of the algorithm.

The main purpose of the present paper is to provide an analysis for a method closely related to that of [12], with an error bound that is independent of stochastic dimension, and a convergence rate faster than that of a simple Monte Carlo method. The setting differs in two ways from [12]: first, the FE method considered here is the standard nodal FE method for elliptic problems, whereas in [12] the mixed FE method was used; and second, the QMC method considered here is a specially designed randomly shifted lattice rule (see (1.12) below), instead of using Sobol’ points as in [12]. (We expect the present analysis can be extended to mixed FEs using results in [14], but do not attempt this here.)

Thus our PDE model (written initially in strong form) is

1.1 $$\begin{aligned} -\nabla \cdot (a({\varvec{x}},\omega ) \nabla u({\varvec{x}},\omega ))&\,=\, f({\varvec{x}})\, \quad \text {for } {\varvec{x}}\in D\subseteq [0,1]^d,\ \ \text {and almost all}\ \ \omega \in \varOmega . \end{aligned}$$

Given a functional $\mathcal {G}$ of *u* with respect to the spatial variable ${\varvec{x}}$, our aim here (as in [12]) is to compute efficiently and accurately $\mathbb {E}[\mathcal {G}(u)]$, the expected value of $\mathcal {G}(u(\cdot , \omega ))$. The (spatial) domain $D \subset \mathbb {R}^d$ ($d = 1,2,3$) in (1.1) is assumed to be a bounded interval ($d=1$), polygon ($d=2$) or Lipschitz polyhedron ($d = 3$), while $\varOmega$ is the set of events in a suitable probability space $(\varOmega ,\mathcal {A},\mathbb {P})$. The solution *u* is required to satisfy the homogeneous Dirichlet condition $u = 0$ on the boundary $\partial D$ of *D*. The spatial domain *D* is allowed to be irregular but we assume for convenience that it can be embedded in the *d*-dimensional unit cube; this is always possible after a suitable affine scaling. (The length-scale of our random field is therefore always considered with respect to the unit cube.) The driving term *f* is for simplicity taken to be deterministic.

We consider the lognormal case where

1.2 $$\begin{aligned} a({\varvec{x}}, \omega ) \,=\, \exp (Z({\varvec{x}},\omega )), \end{aligned}$$

with $Z({\varvec{x}}, \omega )$ a Gaussian random field with prescribed mean $\overline{Z}({\varvec{x}})$ and covariance

1.3 $$\begin{aligned} r_{\mathrm {cov}}({\varvec{x}},{\varvec{x}}') \,:=\, \mathbb {E}[(Z({\varvec{x}},\cdot )-\overline{Z}({\varvec{x}}))(Z({\varvec{x}}',\cdot )-\overline{Z}({\varvec{x}}')], \end{aligned}$$

where the expectation is with respect to the Gaussian measure. Lognormal random fields are commonly used in applications, for example in hydrology (see, e.g., [18] and the references there). Throughout we will assume that *Z* is *stationary* (see, e.g., [1, p. 24]), i.e., its covariance function satisfies

1.4 $$\begin{aligned} r_{\mathrm {cov}}({\varvec{x}},{\varvec{x}}') \,=\, \rho ({\varvec{x}}-{\varvec{x}}'). \end{aligned}$$

Strictly speaking, $\rho$ only needs to be defined on a sufficiently large ball $B(0,{\text {diam}}(D))$ for the prescription above, but as in many applications we assume that it is defined on all of $\mathbb {R}^d$. A particular case that will be discussed extensively, is the Matérn covariance, where $\rho$ is isotropic, i.e., $\rho$ depends on ${\varvec{x}}$ only through its Euclidean length $\Vert {\varvec{x}}\Vert _2$.

In the present paper (1.1) is discretised by piecewise linear finite elements in space, using simplicial meshes with maximum diameter *h*, and a simple low order quadrature rule for suitable approximation of the associated stiffness matrix. In consequence, the only values of the stochastic coefficient $Z({\varvec{x}}, \omega )$ that enter the FE computation are its values at the quadrature points. However, the FE quadrature points will in general be irregularly distributed, and (for refined meshes) very large in number, typically rendering a direct evaluation of the field at the quadrature points prohibitively expensive. It is much more efficient, as explained below, to instead evaluate exactly the realisation of the field at a uniform grid of

$$\begin{aligned} M \,=\, (m_0+1)^d \end{aligned}$$

points ${\varvec{x}}_1,{\varvec{x}}_2,\ldots ,{\varvec{x}}_M$ on the *d*-dimensional unit cube $[0,1]^d$ (containing the domain *D*), with a fixed integer $m_0$ and with grid spacing $h_0 := 1/m_0$. We assume further that $h_0\sim h$. (The extension to general tensor product grids with different mesh sizes in the different coordinate directions is straightforward and not discussed here.) We use *multilinear interpolation* to obtain a sufficiently good approximation of the field $Z({\varvec{x}}, \omega )$ at any other spatial point ${\varvec{x}}\in D$, i.e., we use repeated linear interpolation in each coordinate direction with respect to the vertices of the surrounding grid cell.

At this stage of the algorithm, the output is the approximate FE solution $u_h({\varvec{x}}, \omega )$, which inherits randomness from the input data

$$\begin{aligned} {\varvec{Z}}(\omega ) \,:=\, (Z({\varvec{x}}_1,\omega ), \ldots , Z({\varvec{x}}_M, \omega ))^{\top } \ . \end{aligned}$$

The *M*-vector ${\varvec{Z}}(\omega )$ is a Gaussian random vector with a covariance structure inherited from the continuous field *Z*. Thus it has mean $\overline{{\varvec{Z}}} := (\overline{Z}({\varvec{x}}_1),\ldots ,\overline{Z}({\varvec{x}}_M))^\top$ and a positive definite covariance matrix

1.5 $$\begin{aligned} R \,=\, [\rho ({\varvec{x}}_i-{\varvec{x}}_j)]_{i,j=1}^M. \end{aligned}$$

Because of its finite length, ${\varvec{Z}}(\omega )$ can be expressed exactly (but not uniquely) as a linear combination of a finite number of i.i.d. standard normal random variables, i.e., as

1.6 $$\begin{aligned} {\varvec{Z}}(\omega ) \,=\, B \varvec{Y}(\omega )\ + \ \overline{{\varvec{Z}}}\ , \quad \text {where} \quad \varvec{Y}\sim \mathcal {N}(\varvec{0}, I_{s \times s}). \end{aligned}$$

for some real $M\times s$ matrix *B* with $s\ge M$ satisfying

1.7 $$\begin{aligned} R \,=\, BB^\top . \end{aligned}$$

To see this, note that (1.6) and (1.7) imply

$$\begin{aligned} \mathbb {E}[({\varvec{Z}}-\overline{{\varvec{Z}}})({\varvec{Z}}-\overline{{\varvec{Z}}})^{\top }] \,=\, \mathbb {E}[B\varvec{Y}\varvec{Y}^\top B^{\top }] \,=\, B\,\mathbb {E}[\varvec{Y}\varvec{Y}^\top ]B^{\top }=BB^{\top } \,=\, R. \end{aligned}$$

An efficient computation of a suitable factorisation (1.7) using the extension of *R* to a nested block circulant matrix and then diagonalisation using FFT (the “circulant embedding method”) is described in detail in [5, 6, 9, 12]. It is essential for that approach that the random field is sampled on a uniform grid of points. In a related paper [13], we have analysed certain key properties of the circulant extension and its factorisation, which will be crucial for efficiency and for the dimension independence of the QMC convergence analysis in this paper. Other approaches, such as Cholesky factorisation or direct spectral decomposition, could also be used to find a factorisation of the form (1.7). These alternative approaches have the advantage that they do not require the sample grid to be uniform, but when *M* is large these approaches are likely to be prohibitively expensive. Some ideas of how to overcome this problem using a pivoted Cholesky factorisation or hierarchical matrices can be found in [15] or [4, 10], respectively.

From now on, realisations of the random vector $\varvec{Y}(\omega )$ are denoted by ${\varvec{y}}$. Thus, ${\varvec{y}}$ contains *s* independent realisations of $\mathcal {N}(0,1)$. Hence, if $F: \mathbb {R}^s \rightarrow \mathbb {R}$ is any Lebesgue measurable function then the expected value of $F(\varvec{Y}(\omega ))$ may be written as

1.8 $$\begin{aligned} I_s(F) \ := \int _{\mathbb {R}^s} F({\varvec{y}}) \prod _{j=1}^s \phi (y_j) \, \mathrm {d}{\varvec{y}}\ = \ \int _{{(0,1)^s}} F(\varPhi _s^{-1}({\varvec{v}})) \, \mathrm {d}{\varvec{v}}\ , \end{aligned}$$

where $\phi$ is the one-dimensional standard normal probability density, and $\varPhi _s^{-1}$ is the inverse of the cumulative normal distribution function applied componentwise on $(0,1)^s$. Since $u_h(\cdot , \omega )$ is derived from $\varvec{Y}(\omega )$, we make the notational convention

1.9 $$\begin{aligned} u_h({\varvec{x}}, \omega ) \ = \ u_h({\varvec{x}},{\varvec{y}}), \end{aligned}$$

and so our approximation to $\mathbb {E}[\mathcal {G}(u)]$ is

1.10 $$\begin{aligned} \mathbb {E}[\mathcal {G}(u_h)] \ = \ I_s(F), \quad \text {with}\quad F({\varvec{y}}) = \mathcal {G}(u_h(\cdot , {\varvec{y}})) . \end{aligned}$$

Because $s\ge M$, the integral (1.8) can have very high dimension. However, two important empirical findings in [12] were that (for all the applications considered) the accuracy of the QMC cubature rule based on Sobol$'$ points did not appear to be affected by the size of *s* and that it was always superior to classical Monte Carlo (MC) methods. Successful computations with $s \sim 4\times 10^6$ were reported in [12]. One aim of the present work is to provide a rigorous proof of the independence of the QMC error on the dimension *s* (under appropriate conditions) for a specially designed randomly shifted lattice rule. Furthermore, we will also prove here the superior asymptotic convergence rate of QMC over MC in this setting.

The accuracy of the approximation to $\mathbb {E}[\mathcal {G}(u)]$ depends on the FE mesh diameter *h* (through the FE error and the interpolation error), as well as on the number *n* of QMC points. We analyse convergence with respect to both these parameters. Our first set of theoretical results concern the accuracy with respect to *h*. In particular, for bounded linear functionals $\mathcal {G}$ (with respect to the spatial variable ${\varvec{x}}$) and under suitable assumptions, one result, obtained in Sect. 2.2, is that

1.11 $$\begin{aligned} \vert \,\mathbb {E}\left[ \mathcal {G}(u) - \mathcal {G}({u}_h)\right] \vert \ \le \ C h^{2t}\ , \end{aligned}$$

for some parameter $t \in (0,1]$, determined by the smoothness of realisations of $a({\varvec{x}}, \omega )$, and with a constant *C* independent of *h*. This result differs from that of [21] through the use of interpolation to approximate the random vector ${\varvec{Z}}$.

Then, a substantial part of the paper is concerned with the convergence of the QMC rules for (1.8). In particular, we consider randomly shifted lattice rules,

1.12 $$\begin{aligned} Q_{s,n}({\varvec{\varDelta }}, F) \, := \, \frac{1}{n} \sum _{k=1}^n F \left( \varPhi ^{-1}_s ({\varvec{v}}_k) \right) , \quad \text {with} \quad {\varvec{v}}_k = \mathrm{frac} \left( \frac{k\,{\varvec{z}}}{n} + {\varvec{\varDelta }}\right) , \end{aligned}$$

where ${\varvec{z}}\in \mathbb {N}^s$ is some suitably chosen generating vector, ${\varvec{\varDelta }}\in [0,1]^s$ is a uniformly distributed random shift, and “$\mathrm{frac}$” denotes taking the fractional part of every component in a vector. For the particular integrand $F({\varvec{y}}) := \mathcal {G}({u}_h(\cdot ,{\varvec{y}}))$, we provide in Sect. 3 upper bounds on the error of approximating the integral (1.10) by (1.12). In particular, in Theorem 5, we give sufficient conditions on the matrix *B* appearing in (1.7) for the root-mean-square error to satisfy an estimate of the form:

1.13 $$\begin{aligned} \sqrt{\mathbb {E}_{\varvec{\varDelta }}\left[ |\, I_s(F) - Q_{s,n}({\varvec{\varDelta }}, F) |^2 \right] } \ \le \ \ C_\delta \, n^{-(1-\delta )}\ , \end{aligned}$$

for arbitrary $\delta > 0$. Here, $\mathbb {E}_{\varvec{\varDelta }}$ denotes expectation with respect to the random shift ${\varvec{\varDelta }}$. Moreover, we also provide conditions under which the constant $C_\delta$ in (1.13) is independent of *s*. Our proof of Theorem 5 differs from the corresponding result in [11] because of the use of multilinear interpolation of the random field in space, and because of the different meaning of the parameters $y_j$. The problem is no longer about truncating an infinite Karhunen–Loève expansion, but rather of dealing with a sequence of matrix problems with ever increasing size *s*.

Finally, combining (1.11) and (1.13), the overall error estimate is

$$\begin{aligned} \sqrt{ \mathbb {E}_{\varvec{\varDelta }}\left[ |\,\mathbb {E}[\mathcal {G}(u)] - Q_{s,n}({\varvec{\varDelta }}, F)|^2 \right] } \,\le \, \sqrt{2}\, {C} \, h^{2t} + \sqrt{2}\,C_\delta \, n^{-(1-\delta )} . \end{aligned}$$

Although the algorithm in [12] applies to both linear and nonlinear functionals, our theory at present is restricted to the linear case. In the numerical experiments, we will use the average of *q* different random shifts as our final QMC estimator, bringing the total number of integrand evaluations (i.e., PDE solves) to $N=q\,n$. We compare with a classical Monte Carlo (MC) method for which we use *N* i.i.d. random samples ${\varvec{w}}_k \sim U[0,1)^s$, i.e.,

1.14 $$\begin{aligned} Q^{\text {MC}}_{s,N}(F) \, := \, \frac{1}{N} \sum _{k=1}^N F \left( \varPhi ^{-1}_s ({\varvec{w}}_k) \right) , \quad \text {with} \quad {\varvec{w}}_k \sim U[0,1)^s . \end{aligned}$$

The layout of the paper is as follows. The PDE with random coefficient and its FE approximation with quadrature are described in Sect. 2.1. The estimate (1.11) is proved in Sect. 2.2. The QMC theory is given in Sect. 3. In particular, one of the key results proved in Sect. 3.3 is the upper bound (1.13). A sufficient condition on *B* for this result in the case of circulant embedding is identified in Sect. 3.4. The circulant embedding algorithm is summarised briefly in Sect. 3.4 and we refer to [13] for its theoretical analysis. Numerical experiments are given in Sect. 4, illustrating the performance of the algorithm on PDE problems on an irregular domain with corners and holes in two space dimensions, as well as on the unit cube in three dimensions.

## Finite element implementation and analysis

In Sect. 2.1, we first give the algorithmic details of our practical finite element method, before proving error estimates for this method in Sect. 2.2, in particular Theorem 1 and Corollary 1.

### Model formulation and implementation

We start with (1.1) written pathwise in weak form: seek $u(\cdot ,\omega ) \in V := H^1_0(D)$ such that

2.1 $$\begin{aligned} \mathscr {A}(\omega ; u(\cdot ,\omega ), v) \ = \ \langle f, v\rangle \ \quad \text {for all} \ v \in V\ \text {and for almost all} \ \omega \in \varOmega \ , \end{aligned}$$

where

$$\begin{aligned} \mathscr {A}(\omega ; w,v) \,:=\, \int _D a({\varvec{x}}, \omega )\, \nabla w({\varvec{x}})\cdot \nabla v({\varvec{x}})\,\mathrm {d}{\varvec{x}}\ , \quad w, v \in V , \end{aligned}$$

and $a({\varvec{x}}, \omega )$ is given by (1.2)–(1.4). The norm in *V* is $\Vert v\Vert _V := \Vert \nabla v\Vert _{L^2(D)}$. For simplicity we assume that $f \in L^2(D)$, so that $\langle f,v\rangle$ reduces to the $L^2(D)$ inner product in (2.1). In general, it denotes the duality pairing between *V* and its dual space $V' := H^{-1}(D)$.

To discretise (2.1) in the physical domain *D*, let $\{\mathcal {T}_h\}_{h>0}$ denote a family of conforming, simplicial meshes on *D*, parametrised by the maximum mesh diameter $h := \max _{\tau \in \mathcal {T}_h} {\text {diam}}(\tau )$ with ${\text {diam}}(\tau ) := \max _{{\varvec{x}},{\varvec{x}}' \in \tau } \Vert {\varvec{x}}- {\varvec{x}}'\Vert _2$ . On this mesh, we let $V_h\subset V$ denote the usual finite element space of continuous piecewise linear functions that vanish on $\partial D$. We assume that $\text {dim}(V_h) = \mathcal {O}(h^{-d})$. This includes many locally refined mesh families, including anisotropic refinement in 3D (e.g., [2, 3]). Since any function in $V_h$ has a piecewise constant gradient, we have

2.2 $$\begin{aligned} \mathscr {A}(\omega ; w_h,v_h) \,=\, \sum _{\tau \in \mathcal {T}_h} a_\tau (\omega )\, (\nabla w_h({\varvec{x}})\cdot \nabla v_h({\varvec{x}})) \bigr \vert _\tau \quad \text{ for } \text{ all } v_h, w_h \in V_h\ , \end{aligned}$$

where

2.3 $$\begin{aligned} a_\tau (\omega ) \ := \ \int _\tau a({\varvec{x}}, \omega ) \,\mathrm {d}{\varvec{x}}, \quad \tau \in \mathcal {T}_h\ . \end{aligned}$$

We approximate the required integrals (2.3) using a two-stage interpolation/quadrature process as follows. Recall the uniform grid on the cube containing *D*, with points ${\varvec{x}}_j$ for $j = 1, \ldots , M$, and grid spacing $h_0\sim h$, defined in Sect. 1. Let ${\varvec{x}}\in \overline{D}$ and let $\{{\varvec{t}}_{i,{\varvec{x}}}\}_{i=1}^{2^d} \subset \{{\varvec{x}}_1,\ldots ,{\varvec{x}}_M\}$ be the vertices of the surrounding grid cell labelled in arbitrary order. Since multilinear interpolation is done by repeatedly applying linear interpolation in each coordinate direction, we can write the interpolated value of *g* at ${\varvec{x}}$ as a convex combination of the surrounding vertex values $\{{\varvec{t}}_{i,{\varvec{x}}}\}_{i=1}^{2^d}$, i.e.,

2.4 $$\begin{aligned} \mathcal {I}_{h_0}\left( g;\{{\varvec{x}}_j\}_{j=1}^M\right) ({\varvec{x}}) = \sum _{i=1}^{2^d} w_{i,{\varvec{x}}} \, g({\varvec{t}}_{i,{\varvec{x}}}) ,\quad \text {with}\quad \sum _{i=1}^{2^d} w_{i,{\varvec{x}}} = 1 \;\text { and }\; 0 \le w_{i,{\varvec{x}}} \le 1. \end{aligned}$$

The operator $\mathcal {I}_{h_0}:C(\overline{D})\rightarrow C(\overline{D})$ is linear and satisfies $\mathcal {I}_{h_0}(g;\{{\varvec{x}}_j\}_{j=1}^M)({\varvec{x}}_i) = g({\varvec{x}}_i)$, for every point ${\varvec{x}}_i$ of the uniform grid.

Let us further define an *r*-point quadrature rule on each element $\tau$, which is exact for constant functions, has positive weights $\mu _{\tau ,k} \ge 0$ and only uses quadrature points ${\varvec{x}}_{\tau ,k} \in \tau$, i.e.,

2.5 $$\begin{aligned} Q_\tau (g) := \sum _{k=1}^r \mu _{{\tau },{k}} \, g({\varvec{x}}_{{\tau },{k}}), \quad \text {with} \ \ \sum _{k=1}^r \mu _{{\tau },{k}} = \vert \tau \vert \ \text { and } \ \mu _{\tau ,k} \ge 0. \end{aligned}$$

Here, $|\tau |$ denotes the volume of $\tau$. The quadrature points ${\varvec{x}}_{{\tau },{k}}$ in (2.5) are unrelated to the uniform grid points ${\varvec{x}}_j$ in general. Examples of rules satisfying (2.5) are the centroid, nodal or face-centroid rules.

Using the rule (2.5) to approximate all the integrals (2.3) would require evaluating $a(\cdot , \omega )$ at the union of all the (in general irregularly distributed) quadrature points $\{ {\varvec{x}}_{{\tau },{k}} \}$, which could be costly. We avoid that and compute the field only at the points of the uniform grid. We then interpolate these values using $\mathcal {I}_{h_0}(a(\cdot ,\omega );\{{\varvec{x}}_j\}_{j=1}^M)$ and then approximate $a_\tau (\omega )$ using (2.5). In summary, we approximate the bilinear form in (2.2) by

2.6 $$\begin{aligned} \mathscr {A}_h(\omega ;w_h,v_h) \,:=\, \sum _{\tau \in \mathcal {T}_h} \widehat{a}_\tau (\omega ) \, (\nabla w_h \cdot \nabla v_h) \bigr \vert _\tau \ , \end{aligned}$$

where

2.7 $$\begin{aligned} \widehat{a}_{\tau }(\omega ) \,:=\, Q_\tau \bigl (\mathcal {I}_{h_0}(a(\cdot ,\omega );\{{\varvec{x}}_j\}_{j=1}^M)\bigr ) = (Q_\tau \circ \mathcal {I}_{h_0})(a(\cdot ,\omega )) . \end{aligned}$$

#### Proposition 1

For all $\tau \in \mathcal {T}_h$, there is a sparse positive vector ${\varvec{p}}_\tau =(p_{\tau ,1},\ldots ,p_{\tau ,M}) \in \mathbb {R}^M$ such that

$$\begin{aligned} \widehat{a}_\tau (\omega ) \ = \ \sum _{j=1}^M p_{\tau ,j} \, a({\varvec{x}}_j,\omega ) , \quad \text {and} \quad \widehat{a}_\tau (\omega ) \ \ge \ \vert \tau \vert \, {a}_{\min ,M}(\omega )\,, \quad \text {for all} \ \ \tau \in \mathcal {T}_h\,, \end{aligned}$$

where $$a_{\min ,M}(\omega ) \,:=\, \min _{1\le j\le M} a({\varvec{x}}_j,\omega )$$.

#### Proof

It follows from (2.7) together with (2.4) and (2.5) that

2.8 $$\begin{aligned} \widehat{a}_\tau (\omega ) \,= \, \sum _{k=1}^r \sum _{i=1}^{2^d} \mu _{\tau ,k} \, w_{i,{\varvec{x}}_{\tau ,k}} a({\varvec{t}}_{i,{\varvec{x}}_{\tau ,k}},\omega ). \end{aligned}$$

The second result then follows from the definition of $a_{\min ,M}(\omega )$ and the fact that the coefficients $\mu _{\tau ,k}$ and $w_{i,{\varvec{x}}_{\tau ,k}}$ are all positive and their sum is $|\tau |$. $\square$

Extending the notational convention (1.9), we may thus write our discrete finite element method for (2.1) as the problem of finding $u_h(\cdot , {\varvec{y}})$ which satisfies

2.9 $$\begin{aligned} \mathscr {A}_h({\varvec{y}};u_h(\cdot ,{\varvec{y}}),v_h) \ = \ \langle f,v_h\rangle , \quad \mathrm {for}\ \mathrm {all} \quad v_h \in V_h\, \quad {\varvec{y}}\in \mathbb {R}^s, \end{aligned}$$

where $\mathscr {A}_h({\varvec{y}};w_h,v_h)$ is identified with $\mathscr {A}_h(\omega ;w_h,v_h)$.

### Finite element error analysis

Let us first define some relevant function spaces. Let $C^1(\overline{D})$ denote the space of continuously differentiable functions on *D* with seminorm $\vert \phi \vert _{C^1(\overline{D})} := \sup _{{\varvec{x}}\in \overline{D}} |\nabla \phi ({\varvec{x}})|$. For $\beta \in (0,1)$, let $C^{\beta }({\overline{D}})$ denote the space of Hölder continuous functions on *D* with exponent $\beta$ and let $\vert \phi \vert _{C^\beta (\overline{D})} := \sup _{{\varvec{x}}_1,{\varvec{x}}_2 \in \overline{D}\,:\,{\varvec{x}}_1 \not = {\varvec{x}}_2} |\phi ({\varvec{x}}_1) - \phi ({\varvec{x}}_2)|/\Vert {\varvec{x}}_1 - {\varvec{x}}_2\Vert _2^\beta <\infty$ denote the *Hölder coefficient* which is, in fact, a seminorm. Also let $L^p(\varOmega ,X)$ denote the space of all random fields in a Banach space *X* with bounded *p*th moments over $\varOmega$.

We assume throughout that $Z(\cdot ,\omega ) \in C^\beta (\overline{D})$, for some $\beta \in (0,1]$, $\mathbb {P}$-almost surely. Since $Z({\varvec{x}},\omega )$ is Gaussian, it follows from Fernique’s Theorem that $\Vert a\Vert _{L^p(\varOmega ,C^\beta (\overline{D}))}$ is finite, for all $p \in [1,\infty )$ (see [7]). Moreover, this implies that $\Vert a_{\max } \Vert _{L^p(\varOmega )} < \infty$ and $\Vert 1/a_{\min }\Vert _{L^p(\varOmega )} < \infty$, for all $p \in [1,\infty )$, where $a_{\min }(\omega ) \,:=\, \min _{{\varvec{x}}\in \overline{D}} a({\varvec{x}},\omega )$ and $a_{\max }(\omega ) \,:=\, \max _{{\varvec{x}}\in \overline{D}} a({\varvec{x}},\omega )$.

Models where realisations of $a({\varvec{x}},\omega )$ lack smoothness are often of interest in applications, and a class of coefficients of particular significance is given by the Matérn class with smoothness parameter $\nu \ge 1/2$, described in detail in Example 1. For $\nu \le 1$, realisations are in $C^{\beta }({\overline{D}})$ $\mathbb {P}$-almost surely, for all $0<\beta <\nu$ (see, e.g., [11, 17]).

There are two factors that limit the convergence rate of the finite element error: (i) the regularity of the coefficient field $a(\cdot ,\omega )$ and (ii) the shape of the domain *D*. Since $a(\cdot ,\omega ) \in C^\beta (\overline{D})$, then (if $\partial D$ is smooth enough), we have $u(\cdot , \omega ) \in H^{1+t}(D)$ for all $0\le t \le \beta$. Here, when $\beta < 1$, the loss of $H^2$ regularity is global and the resulting reduction in the finite element convergence rate cannot be corrected by local mesh refinement. On the other hand, the influence of corner or edge singularities can typically be eliminated by suitable local mesh refinement near $\partial D$.

Using the notation in [21, Def. 2.1], let $\lambda _\varDelta (D)$ be the order of the strongest singularity of the Dirichlet-Laplacian on *D*. Then $u(\cdot ,\omega ) \in H^{1+t}(D)$, for all $t \le \lambda _\varDelta (D)$ and $t < \beta$, and $\Vert u\Vert _{L_p(\varOmega ,H^{1+t}(D))}$ is bounded for all $p \in [1,\infty )$ (see [21, Lem. 5.2]). When $\lambda _\varDelta (D) \ge \beta$ uniform mesh refinement leads to a best approximation error that satisfies

2.10 $$\begin{aligned} \inf _{v_h \in V_h} \Vert u(\cdot ,\omega )-v_h\Vert _{V} \le C_{\mathrm{FE}}(\omega ) h^t \,, \quad \text {for all} \ \ t < \beta \,, \end{aligned}$$

with $C_{\mathrm{FE}}(\omega ) \sim \Vert u(\cdot ,\omega ) \Vert _{H^{1+t}(D)}$. When $\lambda _\varDelta (D) < \beta$, (2.10) cannot be achieved by uniform refinement. However, it can be recovered by a suitable local refinement. For example, consider the 2D case where *D* is smooth except for a single reentrant corner with interior angle $\theta > \pi$ and where $W \subset D$ is a local neighbourhood of this corner. Then $\lambda _\varDelta (D) = \pi /\theta$ and $u(\cdot ,\omega ) \in H^{1+t}(D \backslash W)$, for all $t < \beta$, but $u(\cdot ,\omega ) \not \in H^{1+t}(W)$, for $\pi /\theta< t < \beta$. However, by considering the best approximation error over *W* and over $D\backslash W$ separately, we see that it suffices to grade the meshes such that the mesh size is $\mathcal {O}(h^{\beta \theta /\pi })$ near the reentrant corner and $\mathcal {O}(h)$ away from it. This is because

$$\begin{aligned} \inf _{v_h \in V_h} \Vert u(\cdot ,\omega )-v_h\Vert _{V}&\le C_1 \Vert u(\cdot ,\omega )\Vert _{H^{1+t}(D \backslash W)} h^t \\&\quad + C_2 \Vert u(\cdot ,\omega )\Vert _{H^{1+\lambda _\varDelta (D)}(D)} (h^{\beta \theta /\pi })^{\lambda _\varDelta (D)} \end{aligned}$$

for all $0<t<\beta$. Such a mesh grading can often be achieved while retaining the desired complexity estimate $\text {dim}(V_h) \le C h^{-2}$ (e.g., [20]).

Thus, using similar techniques to those in the proof of [21, Lem. 5.2] it can be shown that (2.10) holds with $C_{\mathrm{FE}}(\omega ) \sim \Vert u(\cdot ,\omega )\Vert _{H^{1+t}(D \backslash W)} + \Vert u(\cdot ,\omega )\Vert _{H^{1+\lambda _\varDelta (D)}(D)}$, for all $t < \beta$. The case of multiple reentrant corners can be treated in an identical fashion. Analogous but more complicated (anisotropic) refinement is needed in 3D, especially in the presence of edge-singularities (e.g., [2, 3]). In practice, such local refinements can be constructed adaptively. The important observation here is that the locally refined mesh needs to be constructed only once for $\exp (\overline{Z}(\cdot ))$ (or for one sample of *a*), since the boundary singularities will be the same for all samples.

We start our analysis by estimating the error in approximating $a_\tau (\omega )$ by $\widehat{a}_\tau (\omega )$.

#### Lemma 1

Assume $a(\cdot ,\omega ) \in C^\beta (\overline{D})$ for some $\beta \in (0,1]$. Furthermore, for $h_0\sim h$ and for $\tau \subseteq D$ with ${\text {diam}}(\tau ) \le h$, let $\mathcal {I}_{h_0}$ and $Q_\tau$ be as defined in (2.4) and (2.5), respectively. Then, with $a_\tau (\omega )$ and $\widehat{a}_\tau (\omega )$ given by (2.3) and (2.7),

$$\begin{aligned} \vert a_\tau (\omega ) - \widehat{a}_\tau (\omega ) \vert \,\le \, |\tau | \, h^\beta \, \gamma ^\beta \, |a(\cdot ,\omega )|_{C^\beta (\overline{D})} , \end{aligned}$$

with $\gamma = 1 + \sqrt{d}\,(h_0/h)$. If the quadrature points all lie on the regular grid and we do not need interpolation, we may take $\gamma = 1$.

#### Proof

Using the fact that $a(\cdot ,\omega )$ is continuous, the integral mean value theorem asserts the existence of an ${\varvec{x}}_\tau ^* \in \tau$ such that

$$\begin{aligned} a_\tau (\omega ) \,=\, |\tau | \, a({\varvec{x}}_\tau ^*,\omega ) \,=\, \sum _{k=1}^r \sum _{i=1}^{2^d} \mu _{\tau ,k} \, w_{i,{\varvec{x}}_{\tau ,k}} \, a({\varvec{x}}_\tau ^*,\omega ) , \end{aligned}$$

where we used (2.4) and (2.5). Then it follows from (2.8) that

$$\begin{aligned} \left| a_\tau (\omega ) - \widehat{a}_\tau (\omega )\right|&\,=\, \Bigg | \sum _{k=1}^r \sum _{i=1}^{2^d} \mu _{\tau ,k} \, w_{i,{\varvec{x}}_{\tau ,k}} \left( a({\varvec{x}}_\tau ^*,\omega ) - a({\varvec{t}}_{i,{\varvec{x}}_{\tau ,k}},\omega )\right) \Bigg |\\&\,\le \, \sum _{k=1}^r \sum _{i=1}^{2^d} \mu _{\tau ,k} \, w_{i,{\varvec{x}}_{\tau ,k}} \left| a({\varvec{x}}_\tau ^*,\omega ) - a({\varvec{t}}_{i,{\varvec{x}}_{\tau ,k}},\omega )\right| \\&\,\le \, \sum _{k=1}^r \sum _{i=1}^{2^d} \mu _{\tau ,k} \, w_{i,{\varvec{x}}_{\tau ,k}}\, \Vert {\varvec{x}}_\tau ^*-{\varvec{t}}_{i,{\varvec{x}}_{\tau ,k}}\Vert _2^\beta \, |a(\cdot ,\omega )|_{C^\beta (\overline{D})}\\&\,\le \, \sum _{k=1}^r \sum _{i=1}^{2^d} \mu _{\tau ,k} \, w_{i,{\varvec{x}}_{\tau ,k}} \left( \Vert {\varvec{x}}_\tau ^*- {\varvec{x}}_{\tau ,k}\Vert _2 \right. \\&\quad \left. +\, \Vert {\varvec{x}}_{\tau ,k} - {\varvec{t}}_{i,{\varvec{x}}_{\tau ,k}}\Vert _2\right) ^\beta |a(\cdot ,\omega )|_{C^\beta (\overline{D})}\\&\,\le \, |\tau | \, (h+\sqrt{d}h_0)^\beta \, |a(\cdot ,\omega )|_{C^\beta (\overline{D})}\,. \end{aligned}$$

In the last step we used the fact that the distance between a point in a cell of the regular grid and a vertex of that cell is at most $\sqrt{d}\,h_0$. If the quadrature points all lie on the regular grid then the second term can be omitted. This completes the proof. $\square$

#### Theorem 1

Suppose that $Z(\cdot ,\omega ) \in C^\beta (\overline{D})$ for some $\beta \in (0,1)$ and suppose that there exists a family $\{\mathcal {T}_h\}_{h>0}$ of conforming, simplicial meshes on *D* such that (2.10) holds with $\text {dim}(V_h) \le C h^{-d}$. Let $\mathcal {I}_{h_0}$ and $Q_\tau$ be defined in (2.4) and (2.5), respectively. Then, we have $\mathbb {P}$-almost surely

$$\begin{aligned} \Vert u(\cdot ,\omega ) - u_h(\cdot ,\omega )\Vert _{V} \ \le \ C_{{\mathcal {I}Q}}(\omega ) \, h^t\,, \quad \text {for all} \ \ t < \beta , \end{aligned}$$

with $C_{{\mathcal {I}Q}}$ a positive random variable that satisfies $\mathbb {E}[C_{{\mathcal {I}Q}}^p] < \infty$, for all $p \in [1,\infty )$.

If $Z(\cdot ,\omega ) \in C^1(\overline{D})$ and (2.10) also holds for $t=1$, then

$$\begin{aligned} \Vert u(\cdot ,\omega ) - u_h(\cdot ,\omega )\Vert _{V} \ \le \ C_{{\mathcal {I}Q}}(\omega ) \, h. \end{aligned}$$

#### Proof

The proof follows that of [7, Prop. 3.13]. First, using Lemma 1 and the fact that $\nabla v_{h,\tau } := \nabla v_h \vert _\tau$ is constant, for all piecewise linear finite element functions $v_h \in V_h$, as well as applying the Cauchy–Schwarz inequality in the last step we obtain the estimate

$$\begin{aligned} \vert \mathscr {A}(\omega ; w_h, v_h) - \mathscr {A}_h(\omega ; w_h, v_h) \vert= & {} \sum _{\tau \in \mathcal {T}_h} \left| a_\tau (\omega ) - \widehat{a}_{\tau } (\omega )\right| \, \big \vert (\nabla w_{h} \cdot \nabla v_{h})\vert _\tau \big \vert \\\le & {} h^\beta \, \gamma ^\beta \, | a(\cdot , \omega ) |_{C^\beta (\overline{D})} \sum _{\tau \in \mathcal {T}_h} \vert \tau \vert \, \big \vert \nabla w_{h,\tau } \cdot \nabla v_{h,\tau } \big \vert \\\le & {} h^\beta \, \gamma ^\beta \, | a(\cdot , \omega ) |_{C^\beta (\overline{D})} \, \Vert v_h \Vert _{V} \Vert w_h \Vert _{V} \, . \end{aligned}$$

Now, using this bound in Strang’s First Lemma (cf. [7, Lem. 3.12]), we can write

2.11 $$\begin{aligned}&\Vert u(\cdot ,\omega )-u_h(\cdot ,\omega )\Vert _{V} \nonumber \\&\quad \le \inf _{v_h \in V_h} \Bigg \{ \left( 1+\frac{a_{\mathrm {max}}(\omega )}{a_{\mathrm {min}}(\omega )}\right) \, \Vert u(\cdot ,\omega )-v_h\Vert _{V} + h^\beta \gamma ^\beta \, \frac{|a(\omega )|_{\mathcal {C}^{\beta }(\overline{D})}}{a_{\mathrm {min}}(\omega )} \, \Vert v_h\Vert _{V} \Bigg \}\, . \end{aligned}$$

Since

$$\begin{aligned} \Vert v_h\Vert _{V} \le \Vert u(\cdot ,\omega )-v_h\Vert _{V} + \Vert u(\cdot ,\omega )\Vert _{V} \le \Vert u(\cdot ,\omega )-v_h\Vert _{V} + \frac{\Vert f\Vert _{L^2(D)}}{a_{\mathrm {min}}(\omega )} \, , \end{aligned}$$

we can combine (2.11) with (2.10) to establish the result.

The fact that the constant $C_{{\mathcal {I}Q}}(\omega )$ in the above bounds has bounded moments of any (finite) order is a consequence of our assumptions that $Z({\varvec{x}},\omega )$ is Gaussian and that $Z(\cdot ,\omega ) \in \mathcal {C}^{\beta }(\overline{D})$. As stated above, it can be proved as in [7] via Fernique’s Theorem. $\square$

An $\mathcal {O}(h^{2t})$ bound on the $L^2$-norm of the error follows via the well-known Aubin–Nitsche trick (cf. [7, Cor. 3.10]). We omit this and finish the section with an error bound for linear functionals $\mathcal {G}$ of *u*, which we have already stated in (1.11).

#### Corollary 1

Let $\mathcal {G}$ be a bounded linear functional on $L^2(D)$. Then, under the assumptions of Theorem 1, there exists a constant $C > 0$ independent of *h* and *u* such that

$$\begin{aligned} \mathbb {E}\big [\vert \mathcal {G}(u) - \mathcal {G}(u_h) \vert \big ] \le C h^{2t}, \quad \text {for all} \ \ t < \beta \,. \end{aligned}$$

For $\beta = 1$, we get $\mathbb {E}\big [\vert \mathcal {G}(u) - \mathcal {G}(u_h) \vert \big ] \le C h^{2}$.

#### Proof

The proof follows, as in [21, Lem. 3.3], from Hölder’s inequality using the fact that Theorem 1 applies verbatim also to the FE error $\Vert z(\cdot ,\omega )-z_h(\cdot ,\omega )\Vert _V$ for the dual problem $\mathscr {A}(\omega ; v, z(\cdot ,\omega )) = \mathcal {G}(v)$, for all $v \in V$. $\square$

Using the techniques in [21, §3], this corollary can be extended in a straightforward way also to higher order moments of the error or to functionals $\mathcal {G}$ of *u* that are random, nonlinear or bounded only on a subspace of $L^2(D)$. In summary, we have provided in this section a recipe for extending all results of [21, §3] to general meshes, with the random field being sampled on a regular grid and then interpolated onto the finite element grid.

## QMC error analysis

The QMC theory for integrals of the form (1.8) is set in a special weighted Sobolev space. Provided the integrand lies in this space, we obtain an estimate for the root mean square error when a specially chosen, randomly shifted lattice rule (1.12) is used to approximate (1.8). The cost for explicitly constructing a good rule tailored to our analysis with *n* points in *s* dimensions grows log-linearly in *n* and quadratically in *s* (cf. Remark 1 below). However, applying the rule is essentially as cheap as obtaining samples from a random number generator, see, e.g., [16, §7]. Full details of the convergence theory are in other sources, e.g., [11, 16], so we will be brief here.

Later in this section we use this theory to estimate the error when the rule is applied to the particular *F* given in (1.10). We assume first that the random field *Z* is sampled by employing the quite general factorisation (1.7) of the covariance matrix *R*. Later, in Sect. 3.4 we will discuss the case when this is done by circulant embedding.

### Abstract convergence result and proof strategy

The relevant weighted Sobolev norm is defined as:

3.1 $$\begin{aligned} \Vert F\Vert _{s,{\varvec{\gamma }}}^2 \,:=\, \sum _{\mathfrak {u}\subseteq \{1:s\}} \frac{J_{\mathfrak {u}}(F)}{\gamma _\mathfrak {u}}, \end{aligned}$$

where

$$\begin{aligned} J_{\mathfrak {u}} (F) \,:= & {} \, \int _{\mathbb {R}^{|\mathfrak {u}|}} \bigg ( \int _{\mathbb {R}^{s-|\mathfrak {u}|}} \frac{\partial ^{|\mathfrak {u}|} F}{\partial {\varvec{y}}_\mathfrak {u}}({\varvec{y}}_\mathfrak {u};{\varvec{y}}_{\{1:s\}\setminus \mathfrak {u}})\!\! \prod _{j\in \{1:s\}\setminus \mathfrak {u}} \phi (y_j)\,\mathrm {d}{\varvec{y}}_{\{1:s\}\setminus \mathfrak {u}} \bigg )^2\\&\times \prod _{j\in \mathfrak {u}} \psi _j^2(y_j) \,\mathrm {d}{\varvec{y}}_\mathfrak {u}\,. \end{aligned}$$

Here, $\{1 \! : \! s\}$ denotes the set $\{1,2,\ldots ,s\}$, $\frac{\partial ^{|\mathfrak {u}|}F}{\partial {\varvec{y}}_\mathfrak {u}}$ denotes the mixed first order derivative with respect to the “active” variables $y_j$ with $j\in \mathfrak {u}$, ${\varvec{y}}_{\{1:s\}\setminus \mathfrak {u}}$ denotes the “inactive” variables $y_j$ with $j\notin \mathfrak {u}$, and $\phi$ is the univariate normal probability density (see (1.8)). The remaining ingredients in (3.1) are the *weight parameters* $\gamma _\mathfrak {u}$ and the *weight functions* $\psi _j$, which are used, respectively, to moderate the relative importance of the derivatives of *F* with respect to ${\varvec{y}}_\mathfrak {u}$ and to control the behaviour of these derivatives asymptotically as $\Vert {\varvec{y}}\Vert _\infty \rightarrow \infty$. As in [11], we shall restrict ourselves to the choice

3.2 $$\begin{aligned} \psi _j^2(y_j) \,=\, \exp (-2\,\alpha _j\, |y_j|)\;, \quad \text{ for } \text{ some } \quad \alpha _j > 0 \ . \end{aligned}$$

The following result is then essentially [11, Thm. 15] (see also [16]).

#### Theorem 2

Suppose $\Vert F \Vert _{s, {\varvec{\gamma }}} < \infty$ and *n* is a power of a prime. Then, a generating vector ${\varvec{z}}\in \mathbb {N}^s$ for a randomly shifted lattice rule (1.12) can be constructed so that the root mean square error in applying (1.12) to (1.8) satisfies

3.3 $$\begin{aligned} \sqrt{\mathbb {E}_{\varvec{\varDelta }}\left[ |I_s(F) - Q_{s,n}({\varvec{\varDelta }},F)|^2 \right] } \,\le \, \left( \frac{2}{n}\right) ^{1/(2\kappa )} \, \widetilde{C}_s({\varvec{\gamma }}, {\varvec{\alpha }}, \kappa ) \ \Vert F\Vert _{s,{\varvec{\gamma }}}\,, \end{aligned}$$

for all $\kappa \in (1/2,1]$, where3.4
3.5and $\zeta$ is the Riemann zeta function.

A method for constructing the vector ${\varvec{z}}$ one component at a time is described in [19] for weights ${\varvec{\gamma }}_\mathfrak {u}$ of a special form, see Remark 1 below.

In the remainder of this section we shall apply this theory to the function *F* given in (1.10). The main result is Theorem 5. It is obtained in the steps summarised as follows.

1. By differentiating the parametrised discrete weak form (2.9), we estimate the norms $\Vert \left( \partial ^{\vert \mathfrak {u}\vert } u_h/\partial {\varvec{y}}_\mathfrak {u}\right) (\cdot ,{\varvec{y}}) \Vert _V$ for any $\mathfrak {u}\in \{1:s\}$. The estimate (which uses an induction argument over the set of all partial derivatives of $u_h(\cdot , {\varvec{y}})$) is given in Theorem 3 and involves the quantities
  3.6 $$\begin{aligned} b_j \,:=\, \Vert \mathbf {B}_j\Vert _\infty , \quad j=1,\ldots ,s, \end{aligned}$$
  where $\mathbf {B}_j$ is the *j*th column of the $M\times s$ matrix *B* introduced in (1.7). We let ${\varvec{b}}\in \mathbb {R}^s$ be the vector $(b_1,\ldots ,b_s)^\top$.
2. Using the result from Step 1 and the linearity of $\mathcal {G}$, we estimate $\Vert F\Vert _{s, {\varvec{\gamma }}}$ in Theorem 4. The shape parameters $\alpha _j$ from (3.2) are constrained to be in the range $\alpha _j>b_j$. The precise values of $\alpha _j$ are arbitrary at this point, and so are the values of the weight parameters $\gamma _\mathfrak {u}$.
3. We substitute the result from Step 2 into the right hand side of (3.3) to obtain an error bound for this particular *F*, and we choose the weight parameters $\gamma _\mathfrak {u}$ and then the shape parameters $\alpha _j$ to minimise this bound. The end result, Theorem 5, is a convergence estimate with order $\mathcal {O}(n^{-1/(2\kappa )})$, valid for $\kappa \in (1/2,1]$, and with the implied constant depending on the sum $\sum _{j=1}^s b_j^{2\kappa /(1+\kappa )}$.

The theory (in particular Theorem 5) is essentially independent of the choice of factorisation (1.7). In Sects. 3.4 and 4 we then focus on the circulant embedding approach.

### Regularity of *F*

In this subsection it is helpful to introduce more general partial derivatives than those mixed first order derivatives which appear in (3.1). Thus for any multiindex ${\varvec{\nu }}\in \mathbb {N}^s$ with order $\vert {\varvec{\nu }}\vert = \sum \nu _j$, we let $\partial ^{{\varvec{\nu }}}$ denote the corresponding mixed derivative. For any multiindex ${\varvec{\nu }}\in \mathbb {N}^s$ and any vector ${\varvec{c}}\in \mathbb {R}^s$ we also write ${\varvec{c}}^{\varvec{\nu }}= \textstyle \prod _{j=1}^s c_j^{\nu _j}$.

#### Theorem 3

For any ${\varvec{y}}\in \mathbb {R}^s$, any $f\in V'$, and for any multiindex ${\varvec{\nu }}\in \mathbb {N}^s$, the solution $u_h(\cdot ,{\varvec{y}})$ of (2.9) satisfies

$$\begin{aligned} \Vert \partial ^{{\varvec{\nu }}} u_h(\cdot ,{\varvec{y}}) \Vert _V \,\le \, | {\varvec{\nu }}|! \bigg ( \frac{{\varvec{b}}}{\log 2} \bigg )^{\varvec{\nu }}\frac{1}{{a}_{\min ,M}({\varvec{y}})} \Vert f\Vert _{V'} \;, \end{aligned}$$

where ${\varvec{b}}= (b_1,\ldots ,b_s)^\top$ is defined in (3.6) and $a_{\min ,M} ({\varvec{y}}) = \min _{1\le i\le M} a({\varvec{x}}_i, {\varvec{y}})$.

#### Proof

The proof is similar to that of [11, Thm. 14], but there are some differences due to the fact that we are working with the FE discretisation (2.9) with quadrature and interpolation, and because of the finiteness of the ‘expansion’ (1.6). (In [11] an infinite KL expansion was used in the context of the continuous problem.)

To simplify the proof we introduce the ${\varvec{y}}$-dependent discrete norm $|||\cdot |||$ on $V_h$:

3.7 $$\begin{aligned} |||v_h |||_{{\varvec{y}}}^2 \ := \ \sum _{\tau \in \mathcal {T}_h} \widehat{a}_\tau ({\varvec{y}}) \left. |\nabla v_h|^2 \right| _\tau \ , \quad v_h \in V_h\ , \end{aligned}$$

with $\hat{a}_\tau ({\varvec{y}})=\hat{a}_\tau (\omega )$ given by (2.7). Then we have $|||v_h |||_{{\varvec{y}}}^2 = \mathscr {A}_h({\varvec{y}};v_h,v_h)$, see (2.6). Since we consider piecewise linear finite elements, for $v_h\in V_h$ we have that $\nabla v_h$ is piecewise constant on each element $\tau$.

We first prove by induction on $|{\varvec{\nu }}|$ that the solution $u_h(\cdot , {\varvec{y}})$ of (2.9) satisfies

3.8 $$\begin{aligned} |||\partial ^{{\varvec{\nu }}}u_h(\cdot , {\varvec{y}}) |||_{{\varvec{y}}} \&\,\le \, \varLambda _{|{\varvec{\nu }}|}\, {\varvec{b}}^{{\varvec{\nu }}}\, |||u_h(\cdot , {\varvec{y}}) |||_{{\varvec{y}}} \ , \quad {\varvec{y}}\in \mathbb {R}^s \ , \end{aligned}$$

where the sequence $(\varLambda _n)_{n\ge 0}$ is defined recursively by

$$\begin{aligned} \varLambda _0 \,:=\, 1 \quad \text {and} \quad \varLambda _n \,:=\, \sum _{i=0}^{n-1} \left( {\begin{array}{c}n\\ i\end{array}}\right) \varLambda _i, \quad \text { for all} \quad n\ge 1 \ . \end{aligned}$$

Clearly (3.8) holds for $|{\varvec{\nu }}| = 0$. For ${\varvec{\nu }}\ne {\varvec{0}}$, we differentiate (2.9), using the multivariate Leibniz rule to obtain (since the right-hand side is independent of ${\varvec{y}}$),

3.9 $$\begin{aligned} \sum _{{\varvec{m}}\le {\varvec{\nu }}} \left( {\begin{array}{c}{\varvec{\nu }}\\ {\varvec{m}}\end{array}}\right) \sum _{\tau \in \mathcal {T}_h} \big (\partial ^{{\varvec{\nu }}- {\varvec{m}}} \widehat{a}_\tau ({\varvec{y}}) \big ) \big (\nabla \partial ^{{\varvec{m}}} u_h(\cdot ,{\varvec{y}}) \cdot \nabla v_h\big )\big |_\tau \ = \ 0 , \quad \text {for all} \quad v_h \in V_h . \end{aligned}$$

Now inserting $v_h = \partial ^{{\varvec{\nu }}} u_h(\cdot ,{\varvec{y}})$ into (3.9), keeping the term with ${\varvec{m}}= {\varvec{\nu }}$ in the outer sum on the left-hand side and moving the remaining terms to the right-hand side, we have

$$\begin{aligned}&|||\partial ^{{\varvec{\nu }}} u_h(\cdot ,{\varvec{y}})|||_{{\varvec{y}}}^2 \,=\, - \sum _{{\mathop {\scriptstyle {{\varvec{m}}\ne {\varvec{\nu }}}}\limits ^{\scriptstyle {{\varvec{m}}\le {\varvec{\nu }}}}}} \left( {\begin{array}{c}{\varvec{\nu }}\\ {\varvec{m}}\end{array}}\right) \sum _{\tau \in \mathcal {T}_h} \big (\partial ^{{\varvec{\nu }}- {\varvec{m}}}\widehat{a}_\tau ({\varvec{y}}) \big ) \big (\nabla \partial ^{{\varvec{m}}} u_h(\cdot ,{\varvec{y}}) \cdot \nabla \partial ^{{\varvec{\nu }}} u_h(\cdot ,{\varvec{y}}) \big )\big |_\tau \nonumber \\&\quad \le \sum _{{\mathop {\scriptstyle {{\varvec{m}}\ne {\varvec{\nu }}}}\limits ^{\scriptstyle {{\varvec{m}}\le {\varvec{\nu }}}}}} \left( {\begin{array}{c}{\varvec{\nu }}\\ {\varvec{m}}\end{array}}\right) \left( \max _{\tau \in \mathcal {T}_h} \left| \frac{\partial ^{{\varvec{\nu }}- {\varvec{m}}} \widehat{a}_\tau ({\varvec{y}})}{\widehat{a}_\tau ({\varvec{y}})}\right| \right) \, \sum _{\tau \in \mathcal {T}_h} \widehat{a}_\tau ({\varvec{y}})\, \big | \big (\nabla \partial ^{{\varvec{m}}} u_h(\cdot ,{\varvec{y}}) \cdot \nabla \partial ^{{\varvec{\nu }}} u_h(\cdot ,{\varvec{y}})\big ) \big |_\tau \big | . \end{aligned}$$

Then, after an application of the Cauchy–Schwarz inequality and a cancellation, we obtain

3.10 $$\begin{aligned} |||\partial ^{{\varvec{\nu }}} u_h(\cdot ,{\varvec{y}})|||_{{\varvec{y}}}&\,\le \, \sum _{{\mathop {\scriptstyle {{\varvec{m}}\ne {\varvec{\nu }}}}\limits ^{\scriptstyle {{\varvec{m}}\le {\varvec{\nu }}}}}} \left( {\begin{array}{c}{\varvec{\nu }}\\ {\varvec{m}}\end{array}}\right) \left( \max _{\tau \in \mathcal {T}_h} \left| \frac{\partial ^{{\varvec{\nu }}- {\varvec{m}}} \widehat{a}_\tau ({\varvec{y}})}{\widehat{a}_\tau ({\varvec{y}})}\right| \right) \, |||\partial ^{{\varvec{m}}} u_h(\cdot ,{\varvec{y}})|||_{{\varvec{y}}} \ . \end{aligned}$$

To estimate (3.10), we have from Proposition 1 that $\widehat{a}_\tau ({\varvec{y}}) = \sum _{i=1}^M p_{\tau ,i} \,a({\varvec{x}}_i,{\varvec{y}})$ with all $p_{\tau ,i}\ge 0$, and we recall from (1.2) and (1.6) that $a({\varvec{x}}_i,{\varvec{y}}) = \exp (\sum _{j=1}^s B_{i,j}y_j + \overline{Z}_i)$. Then, noting that $a({\varvec{x}}_i,{\varvec{y}})\ge 0$ it is easy to see that, for any multiindex ${\varvec{\nu }}$,

$$\begin{aligned} |\partial ^{{\varvec{\nu }}} a({\varvec{x}}_i,{\varvec{y}})| \ = \ a({\varvec{x}}_i,{\varvec{y}}) \prod _{j=1}^{s} |B_{i,j}^{\nu _j}| \, \ \le \ a({\varvec{x}}_i,{\varvec{y}})\,{\varvec{b}}^{\varvec{\nu }}\ , \end{aligned}$$

which leads to $\vert \partial ^{{\varvec{\nu }}} \widehat{a}_\tau ({\varvec{y}}) \vert \le \widehat{a}_\tau ({\varvec{y}}) {\varvec{b}}^{\varvec{\nu }}$, and hence

$$\begin{aligned} \max _{\tau \in \mathcal {T}_h} \left| \frac{\partial ^{{\varvec{\nu }}- {\varvec{m}}} \widehat{a}_\tau ({\varvec{y}})}{\widehat{a}_\tau ({\varvec{y}})}\right| \,\le \, {\varvec{b}}^{{\varvec{\nu }}-{\varvec{m}}}\,. \end{aligned}$$

Inserting this into (3.10) we obtain

3.11 $$\begin{aligned} |||\partial ^{{\varvec{\nu }}} u_h(\cdot ,{\varvec{y}})|||_{{\varvec{y}}}&\,\le \, \sum _{{\mathop {\scriptstyle {{\varvec{m}}\ne {\varvec{\nu }}}}\limits ^{\scriptstyle {{\varvec{m}}\le {\varvec{\nu }}}}}} \left( {\begin{array}{c}{\varvec{\nu }}\\ {\varvec{m}}\end{array}}\right) \, {\varvec{b}}^{{\varvec{\nu }}-{\varvec{m}}} |||\partial ^{{\varvec{m}}} u_h(\cdot ,{\varvec{y}})|||_{{\varvec{y}}} \ . \end{aligned}$$

Using (3.11), the estimate (3.8) then follows by induction in exactly the same way as in [11, Thm. 14].

Now, using the definition of the discrete norm (3.7) and the fact that $u_h(\cdot ,{\varvec{y}})$ is the solution of (2.9), we have

3.12 $$\begin{aligned} \left( \min _{\tau \in \mathcal {T}_h}\frac{\widehat{a}_\tau ({\varvec{y}})}{\vert \tau \vert } \right) \Vert u_h(\cdot , {\varvec{y}})\Vert _V^2 \,\le \, |||u_h(\cdot , {\varvec{y}})|||^2_{{\varvec{y}}} = \langle f, u_h(\cdot , {\varvec{y}})\rangle \,\le \, \Vert f \Vert _{V'} \Vert u_h(\cdot , {\varvec{y}})\Vert _V \ . \end{aligned}$$

Hence, using Proposition 1, we conclude that

3.13 $$\begin{aligned} \Vert u_h(\cdot , {\varvec{y}})\Vert _V \,\le \, \frac{\Vert f \Vert _{V'}}{{a}_{\min ,M}({\varvec{y}})} \quad \text{ and }\quad |||u_h(\cdot ,{\varvec{y}})|||_{{\varvec{y}}} \,\le \, \frac{\Vert f \Vert _{V'}}{\sqrt{{a}_{\min ,M}({\varvec{y}})}} \ . \end{aligned}$$

Using the same argument as for the lower bound in (3.12), together with Proposition 1 again, we obtain from (3.8) and (3.13) that

$$\begin{aligned} \sqrt{a_{\min ,M}({\varvec{y}})}\, \Vert \partial ^{{\varvec{\nu }}} u_h(\cdot , {\varvec{y}})\Vert _V \,\le \, |||\partial ^{{\varvec{\nu }}} u_h(\cdot ,{\varvec{y}})|||_{{\varvec{y}}} \,\le \, \varLambda _{|{\varvec{\nu }}|}\,{\varvec{b}}^{\varvec{\nu }}\, \frac{\Vert f \Vert _{V'}}{\sqrt{{a}_{\min ,M}({\varvec{y}})}}. \end{aligned}$$

This together with the estimate $\varLambda _n \ \le n!/(\log 2)^n$ (proved in [11, Thm. 14]) completes the proof. $\square$

We can now use this theorem to show that *F* lies in the weighted Sobolev space characterised by the norm (3.1). We make use of the *s*-dependent quantities

$$\begin{aligned} \Vert {\varvec{b}}\Vert _{p,s} = \Bigg (\sum _{j=1}^s \vert b_j\vert ^p \Bigg )^{1/p}, \quad p > 0\ , \quad \text {and} \quad \Vert {\varvec{b}}\Vert _{\infty ,s} = \max _{j \in \{1:s\}} \vert b_j\vert \ . \end{aligned}$$

#### Theorem 4

Suppose that $\Vert {\varvec{b}}\Vert _{1,s}$ is uniformly bounded with respect to *s*. Suppose also that $\alpha _j > b_j$ for all *j*. Then, for any $f\in V'$ and for any linear functional $\mathcal {G}\in V'$, the integrand $F({\varvec{y}}) = \mathcal {G}(u_h(\cdot ,{\varvec{y}}))$ in (1.10) satisfies

$$\begin{aligned} {\Vert F \Vert _{s, {\varvec{\gamma }}}} \,\le \, C \Bigg (\sum _{\mathfrak {u}\subseteq \{1:s\}} \frac{1}{\gamma _\mathfrak {u}} \left( \frac{\vert \mathfrak {u}\vert !}{(\log 2)^{\vert \mathfrak {u}\vert }}\right) ^2 \prod _{j \in \mathfrak {u}} \frac{\widetilde{b}_j^2}{\alpha _j - b_j}\Bigg )^{1/2}\, , \end{aligned}$$

where *C* is independent of *s* and

$$\begin{aligned} \widetilde{b}_j = \frac{b_j}{2 \exp (b_j^2/2) \varPhi (b_j)}\,, \end{aligned}$$

with $\varPhi$ denoting the univariate standard cumulative normal distribution function.

#### Proof

Using the linearity of $\mathcal {G}$, together with Theorem 3, but replacing the multiindex ${\varvec{\nu }}$ with any set $\mathfrak {u}\subseteq \{1:s\}$ (i.e., restricting to the case where all $\nu _j\le 1$), we obtain the following estimate for the first order partial derivatives of *F* appearing in the norm (3.1),

3.14 $$\begin{aligned} \left| \frac{\partial ^{\vert \mathfrak {u}\vert } F}{\partial {\varvec{y}}_\mathfrak {u}}({\varvec{y}}) \right|&\,\le \, \frac{| \mathfrak {u}|!}{(\log 2)^{\vert \mathfrak {u}\vert }} \bigg (\prod _{j\in \mathfrak {u}} b_j\bigg ) \frac{1}{a_{\min ,M}({\varvec{y}})}\, \Vert f\Vert _{V'}\, \Vert \mathcal {G}\Vert _{V'} \nonumber \\&\,\le \, \frac{| \mathfrak {u}|!}{(\log 2)^{\vert \mathfrak {u}\vert }} \bigg (\exp ({\varvec{b}}^\top \vert {\varvec{y}}\vert ) \prod _{j\in \mathfrak {u}} b_j\bigg ) \bigg (\exp (\Vert \overline{{\varvec{Z}}}\Vert _\infty )\, \Vert f\Vert _{V'}\, \Vert \mathcal {G}\Vert _{V'} \bigg ) \ , \end{aligned}$$

where we used the estimate $a_{\min ,M}({\varvec{y}}) = \min _{1\le i\le M} a({\varvec{x}}_i, {\varvec{y}}) \ge \exp (-\Vert \overline{{\varvec{Z}}}\Vert _\infty )\exp (- {\varvec{b}}^{\mathsf{T}} \vert {\varvec{y}}\vert )$.

Examining the right-hand side of (3.14), we see that the only factor which depends on ${\varvec{y}}$ is $\exp ({\varvec{b}}^\top |{\varvec{y}}|)$. An elementary calculation (see [11, Thm. 16]) shows that

$$\begin{aligned}&\int _{\mathbb {R}^{|\mathfrak {u}|}} \bigg ( \int _{\mathbb {R}^{s-|\mathfrak {u}|}} \bigg (\exp ({\varvec{b}}^\top |{\varvec{y}}|)\prod _{j\in \mathfrak {u}} b_j\bigg ) \prod _{j\in \{1:s\}\setminus \mathfrak {u}} \phi (y_j)\,\mathrm {d}{\varvec{y}}_{\{1:s\}\setminus \mathfrak {u}} \bigg )^2 \prod _{j\in \mathfrak {u}} \psi _j^2(y_j) \,\mathrm {d}{\varvec{y}}_\mathfrak {u}\\&\quad = \Bigg ( \prod _{j\in \{1:s\}\backslash \mathfrak {u}} \left( 2 \varPhi (b_j) \exp (b_j^2/2)\right) ^2 \Bigg ) \Bigg ( \prod _{j \in \mathfrak {u}} \frac{{b}_j^2}{\alpha _j-b_j}\Bigg ) \\&\quad = \Bigg ( \prod _{j\in \{1:s\} } \left( 2 \varPhi (b_j) \exp (b_j^2/2)\right) ^2 \Bigg ) \Bigg ( \prod _{j \in \mathfrak {u}} \frac{{\widetilde{b}}_j^2}{\alpha _j-b_j}\Bigg ) \ . \end{aligned}$$

Since $2\varPhi (b_j)\le 1 + 2b_j/\sqrt{2\pi } < \exp (b_j)$ for all *j*, we have

$$\begin{aligned} \prod _{j\in \{1:s\} } \left( 2 \varPhi (b_j) \exp (b_j^2/2)\right) ^2 \,\le \, \exp (2\Vert {\varvec{b}}\Vert _{1,s} + \Vert {\varvec{b}}\Vert _{2,s}^2)\,. \end{aligned}$$

Thus, it follows from (3.14) and the definition of the norm (3.1) that

$$\begin{aligned} \frac{\Vert F \Vert _{s, {\varvec{\gamma }}}}{\exp (\Vert \overline{{\varvec{Z}}}\Vert _\infty )\, \Vert f\Vert _{V'} \Vert \mathcal {G}\Vert _{V'}}&\,\le \, \exp (2\Vert {\varvec{b}}\Vert _{1,s} + \Vert {\varvec{b}}\Vert _{2,s}^2) \\&\qquad \times \Bigg ( \sum _{\mathfrak {u}\subseteq \{1:s\}} \frac{1}{\gamma _\mathfrak {u}} \left( \frac{\vert \mathfrak {u}\vert !}{(\log 2)^{\vert \mathfrak {u}\vert }}\right) ^2 \prod _{j \in \mathfrak {u}} \frac{\widetilde{b}_j^2}{\alpha _j - b_j}\Bigg )^{1/2}. \end{aligned}$$

The final result, with the constant factor *C* being independent of *s*, is then a consequence of the assumption on ${\varvec{b}}$. $\square$

### Error estimate

In order to obtain a dimension-independent estimate for the QMC method we need a stronger assumption on ${\varvec{b}}$ than that used in Theorem 4. The following theorem shows that under that stronger assumption there is a choice of ${\varvec{\gamma }}$ and ${\varvec{\alpha }}$ which ensures that the QMC error is bounded independently of *s*. The appropriate choice of ${\varvec{\gamma }}$ is of “POD” type, which allows a good generating vector ${\varvec{z}}$ for the QMC rule to be efficiently computed by the “component-by-component” procedure, see Remark 1 below.

#### Theorem 5

Under the assumptions of Theorem 4, let $\kappa \in (1/2,1)$, set $p = 2 \kappa /(1+\kappa )$ and assume in addition that $\Vert {\varvec{b}}\Vert _{p,s}$ is uniformly bounded with respect to *s*. Then there exists a positive constant $C(\kappa )$ depending on $\kappa$ (as well as on $\overline{{\varvec{Z}}}$, *f*, $\mathcal {G}$) such that

3.15 $$\begin{aligned} \sqrt{\mathbb {E}_{\varvec{\varDelta }}[|\,I_s(F) - Q_{s,n}({\varvec{\varDelta }},F)|^2]} \,\le \, C(\kappa ) \, n^{-1/(2\kappa )}\,. \end{aligned}$$

#### Proof

Some parts of the proof are similar to that of [11, Thm. 20], and for these parts we will be brief. We remind the readers that each vector ${\varvec{b}}= (b_1,\ldots ,b_s)^\top$ depends fundamentally on *s*; changing the value of *s* leads to completely different components $b_j$. This is different from the situation in [11] where there is just one infinite sequence ${\varvec{b}}$ that is truncated to *s* terms.

First, combining Theorems 2 and 4 we see that (3.15) holds with $C(\kappa )$ proportional to

$$\begin{aligned}&{C}_s({\varvec{\gamma }}, {\varvec{\alpha }},\kappa ) \,:=\, \widetilde{C}_s({\varvec{\gamma }},{\varvec{\alpha }},\kappa ) \Bigg (\sum _{\mathfrak {u}\subseteq \{1:s\}} \frac{1}{\gamma _\mathfrak {u}} \left( \frac{|\mathfrak {u}|!}{\,{(\log 2)^{|\mathfrak {u}|}}}\right) ^2\, \prod _{j\in \mathfrak {u}} \frac{\widetilde{b}_j^2}{\alpha _j-b_j} \Bigg )^{1/2},\, \end{aligned}$$

with $\widetilde{C}_s({\varvec{\gamma }},{\varvec{\alpha }},\kappa )$ defined in (3.4). Now, we choose the weight parameters ${\varvec{\gamma }}$ to minimise $C_s({\varvec{\gamma }}, {\varvec{\alpha }}, \kappa )$. This minimisation problem was solved in [11, Lem. 18], yielding the solution:

3.16 $$\begin{aligned} \gamma _\mathfrak {u}\, = \, \gamma _\mathfrak {u}^* \,:=\, \bigg ( \bigg (\frac{|\mathfrak {u}|!}{(\log 2)^{|\mathfrak {u}|}}\bigg )^2 \prod _{j\in \mathfrak {u}} \frac{\widetilde{b}_j^2}{(\alpha _j-b_j)\,\varrho (\alpha _j, \kappa )} \bigg )^{1/(1+\kappa )}, \end{aligned}$$

which is of “product and order dependent” (POD) form. With this choice, one can show that

$$\begin{aligned} C_s({\varvec{\gamma }}^*, {\varvec{\alpha }}, \kappa ) \ = \ S_s({\varvec{\alpha }}, \kappa )^{(\kappa +1)/(2 \kappa )}\ , \end{aligned}$$

where

3.17 $$\begin{aligned} {S}_s({\varvec{\alpha }},\kappa ) \,=\, \sum _{\mathfrak {u}\subseteq \{1:s\}} \bigg ( \bigg (\frac{|\mathfrak {u}|!}{(\log 2)^{|\mathfrak {u}|}}\bigg )^2 \, \prod _{j\in \mathfrak {u}} \frac{\varrho ^{1/\kappa }(\alpha _j,\kappa )\,\widetilde{b}_j^2}{\alpha _j-b_j}\bigg )^{\kappa /(1+\kappa )} \,. \end{aligned}$$

It remains to estimate $S_s({\varvec{\alpha }}, \kappa )$. Apart from the constraint $\alpha _j > b_j$, the shape parameters $\alpha _j$ are still free at this stage and so we choose them to minimise the right-hand side of (3.17). This minimisation problem is also solved in [11, Cor. 21]); the solution is

3.18 $$\begin{aligned} \alpha _j \,:=\, \frac{1}{2}\bigg (b_j + \sqrt{b_j^2+1-\frac{1}{2\kappa }}\,\bigg ). \end{aligned}$$

Now, to estimate $S_s({\varvec{\alpha }},\kappa )$, let $b_{\max }$ be an upper bound on $\Vert {\varvec{b}}\Vert _{\infty ,s}$ for all *s* (guaranteed by assumption). Then $b_j \,\le \, b_{\max }$ for all $j=1,\ldots ,s$ and all *s*. For a given value of $\kappa \in (1/2,1]$, let $\alpha _{\max }$ denote the value of (3.18) with $b_j$ replaced by $b_{\max }$. Then we have $\alpha _j \le \alpha _{\max }$ for all $j=1,\ldots ,s$ and all *s*, and

$$\begin{aligned} {\alpha _j - b_j} = \frac{1}{2} \frac{1-1/(2\kappa )}{\sqrt{b_j^2+1-1/(2\kappa )} + b_j} \,&\ge \, \frac{1}{2} \frac{1-1/(2\kappa )}{\sqrt{b_{\max }^2+1-1/(2\kappa )} + b_{\max }} \,\\&= {\alpha _{\max } - b_{\max }}. \end{aligned}$$

Note also that for $\varrho$ defined in (3.5) we have $\varrho (\alpha _j,\kappa ) \le \varrho (\alpha _{\max },\kappa )$ for all *j* and all *s*. Moreover, since $2\exp (b_j^2/2)\varPhi (b_j)\ge 1$, it follows that $\widetilde{b}_j \le b_j$, and so from (3.17) we can conclude that, with $p := 2\kappa /(1+\kappa )$,

$$\begin{aligned} S_s({\varvec{\alpha }},\kappa ) \,\le \, \sum _{\mathfrak {u}\subseteq \{1:s\}} (|\mathfrak {u}|!)^{p} \prod _{j\in \mathfrak {u}} (\tau _{\kappa }\, b_j^2)^{p/2} \ = \ \sum _{\ell = 0}^s (\ell !)^{p} \!\!\sum _{\mathfrak {u}\subseteq \{1:s\},\,|\mathfrak {u}|=\ell }\; \prod _{j \in \mathfrak {u}} (\tau _{\kappa } b_j^2)^{p/2} \;, \end{aligned}$$

where

$$\begin{aligned} \tau _{\kappa } \,:=\, \frac{\varrho ^{1/\kappa }(\alpha _{\max },\kappa )}{(\log 2)^2(\alpha _{\max } - b_{\max })} . \end{aligned}$$

Now using the inequality

$$\begin{aligned} \ell ! \, \sum _{{\mathop {\scriptstyle {|\mathfrak {u}|=\ell }}\limits ^{\scriptstyle {\mathfrak {u}\subseteq \{1:s\}}}}} \prod _{j\in \mathfrak {u}} a_j \le \bigg (\sum _{j=1}^s a_j\bigg )^\ell , \end{aligned}$$

(which holds since for $|\mathfrak {u}| = \ell$ each term $\prod _{j\in \mathfrak {u}} a_j$ from the left-hand side appears in the expansion of the right-hand side exactly $\ell !$ times, but the right-hand side includes other terms) and with *K* denoting the assumed uniform bound on $\Vert {\varvec{b}}\Vert _{p,s}^p$, we obtain

$$\begin{aligned} S_s({\varvec{\alpha }},\kappa ) \,\le \, \sum _{\ell = 0}^s (\ell !)^{p -1} \tau _{\kappa }^{p\ell /2} \bigg (\sum _{j=1}^s b_j^{p} \bigg )^\ell \le \, \sum _{\ell = 0}^\infty (\ell !)^{p -1} \tau _{\kappa }^{p\ell /2} K^\ell \,<\,\infty \,. \end{aligned}$$

The finiteness of the right-hand side follows by the ratio test, on noting that $p<1$. $\square$

#### Remark 1

A generating vector ${\varvec{z}}\in \mathbb {N}^s$ for a randomly shifted lattice rule with *n* points in *s* dimensions that achieves the desired error bound can be constructed using a component-by-component (CBC) algorithm, which goes as follows: (1) Set $z_1 = 1$. (2) For each $k = 2,3,\ldots , s$, choose $z_k$ from the set $\{1\le z\le n-1: \gcd (z,n)=1\}$ to minimise

$$\begin{aligned} E^2_{s,n,k}(z_1,\ldots ,z_k) \,:=\, \sum _{\emptyset \ne \mathfrak {u}\subseteq \{1:k\}} \frac{\gamma _\mathfrak {u}}{n}\sum _{i=1}^n \prod _{j\in \mathfrak {u}}\theta _j\left( \mathrm{frac}\left( \frac{iz_j}{n} \right) \right) , \end{aligned}$$

where the function $\theta _j(x)$ is symmetric around 1 / 2 for $x\in [0,1]$ and can be computed for $x\in [0,1/2]$ by

$$\begin{aligned} \theta _j(x) \,:=\, \frac{x - \frac{1}{2} + \exp (2 \alpha _j^2) \left[ \varPhi (2 \alpha _j) - \varPhi \big (2\alpha _j+\varPhi ^{-1}(x)\big ) \right] }{\alpha _j} -2 \int _{-\infty }^0 \frac{\varPhi (t)^2}{\psi _j(t)^2} \mathrm {d}{t}. \end{aligned}$$

The integral in the above formula for $\theta _j$ only needs to be calculated once, while the general formulation [19, Equation (50)] also has an integral for the first part, which we evaluate explicitly here for our particular choice of $\psi _j$. Note that $\gamma _\mathfrak {u}$ and $\alpha _j$ fundamentally depend on *s* through $b_j$. When (and only when) the algorithm reaches $k=s$, the expression $E^2_{s,n,s}(z_1,\ldots ,z_s)$ is the so-called *squared shift-averaged worst case error*. See [19] for the analysis of an efficient implementation of the algorithm for POD weights (3.16), so that the cost is $\mathcal {O}(sn\log n + s^2n)$ operations using FFT. We refer to the accompanying software of [16] for an implementation.

### QMC convergence in the case of circulant embedding

The circulant embedding technique is a method of computing efficiently the factorisation (1.7), thus yielding a method of sampling the random vector ${\varvec{Z}}$ via (1.6). We describe the process briefly here before verifying the assumptions of Theorem 5. This section is a summary of our results in [13].

The $M = (m_0+1)^d$ points $\{{\varvec{x}}_i: i = 1, \ldots , M\}$ are assumed to be uniformly spaced with spacing $h_0 :=1/m_0$ on a *d*-dimensional grid over the unit cube $[0,1]^d$ enclosing the domain *D*. Using a vector notation, we may relabel the points ${\varvec{x}}_1,\ldots ,{\varvec{x}}_M$ to be indexed by ${\varvec{k}}$ as

$$\begin{aligned} {\varvec{x}}_{{\varvec{k}}} \,:=\, h_0 {\varvec{k}}\quad \text {for}\quad {\varvec{k}}= (k_1, \ldots , k_d) \in \{0 , \ldots , m_0\}^d . \end{aligned}$$

Then it is easy to see that (with analogous vector notation for the rows and columns) the $M \times M$ covariance matrix *R* defined in (1.5) can be written as

3.19 $$\begin{aligned} R_{{\varvec{k}}, {\varvec{k}}'} \,=\, \rho \big (h_0 ({\varvec{k}}-{\varvec{k}}') \big ), \quad {\varvec{k}}, {\varvec{k}}' \in \{ 0, \ldots , m_0\}^d . \end{aligned}$$

If the vectors ${\varvec{k}}$ are enumerated in lexicographical ordering, then we obtain a nested block Toeplitz matrix where the number of nested levels is the physical dimension *d*.

We extend *R* to a nested block circulant matrix $R^{\mathrm {ext}}$. To do this, it is convenient to extend to the infinite grid:

$$\begin{aligned} {\varvec{x}}_{{\varvec{k}}} \,:=\, h_0 {\varvec{k}}\quad \text {for}\quad {\varvec{k}}\in \mathbb {Z}^d . \end{aligned}$$

Then, to define $R^{\mathrm {ext}}$, we consider an enlarged cube $[0,{\ell }]^d$ of edge length ${\ell }:=mh_0\ge 1$ with integer $m\ge m_0$. We assume that $m_0$ (and hence $h_0$) is fixed and we enlarge *m* (or equivalently $\ell$) as appropriate. We introduce a $2 {\ell }$-periodic map on $\mathbb {R}$ by specifying its action on $[0,2 {\ell }]$:

$$\begin{aligned} \varphi (x) \,:=\, {\left\{ \begin{array}{ll} x &{} \text {if}\quad 0\,\le \, x \,\le \, {\ell },\\ 2{\ell }- x &{} \text {if}\quad {\ell }\,\le \, x \,<\, 2{\ell }. \end{array}\right. } \end{aligned}$$

Now we apply this map elementwise and define an extended version $\rho ^{\mathrm {ext}}$ of $\rho$ as follows:

$$\begin{aligned} \rho ^{\mathrm {ext}}({\varvec{x}}) \,:=\, \rho (\varphi (x_1),\ldots ,\varphi (x_d)) ,\quad {\varvec{x}}\in \mathbb {R}^d . \end{aligned}$$

Note that $\rho ^{\mathrm {ext}}$ is $2{\ell }$-periodic in each coordinate direction and $\rho ^{\mathrm {ext}}({\varvec{x}}) = \rho ({\varvec{x}})$ when ${\varvec{x}}\in [0,{\ell }]^d$. Then $R^{\mathrm {ext}}$ is defined to be the $s\times s$ symmetric nested block circulant matrix with $s = (2m)^d$, defined, analogously to (3.19), by

3.20 $$\begin{aligned} R^{\mathrm {ext}}_{{\varvec{k}}, {\varvec{k}}'}\,=\, \rho ^\mathrm{{ext}}\big ( h_0 ({\varvec{k}}- {\varvec{k}}') \big ), \quad {\varvec{k}}, {\varvec{k}}' \in \{0, \ldots , 2m-1\}^d . \end{aligned}$$

It follows that *R* is the submatrix of $R^{\mathrm {ext}}$ in which the indices are constrained to lie in the range ${\varvec{k}}, {\varvec{k}}' \in \{ 0,\ldots , m_0\}^d$. Since $R^{\mathrm {ext}}$ is nested block circulant, it is diagonalisable by FFT. The following theorem is taken from [12]:

#### Theorem 6

$R^{\mathrm {ext}}$ has the spectral decomposition:

$$\begin{aligned} \ R^{\mathrm {ext}}\ =\ Q^{\mathrm {ext}}\varLambda ^{\mathrm {ext}}Q^{\mathrm {ext}}, \end{aligned}$$

where $\varLambda ^{\mathrm {ext}}$ is the diagonal matrix containing the eigenvalues of $R^{\mathrm {ext}}$, which can be obtained by $\sqrt{s}$ times the Fourier transform on the first column of $R^{\mathrm {ext}}$, and $Q^{\mathrm {ext}}= \mathfrak {Re}(\mathcal {F}) + \mathfrak {Im}(\mathcal {F})$ is real symmetric, with

$$\begin{aligned} \mathcal {F}_{{\varvec{k}}, {\varvec{k}}'} = \frac{1}{\sqrt{s}} \exp \left( {2 \pi } \mathrm {i}\frac{{\varvec{k}}' \cdot {\varvec{k}}}{{2 m}} \right) \end{aligned}$$

denoting the *d*-dimensional Fourier matrix. If the eigenvalues of $R^{\mathrm {ext}}$ are all non-negative then the required *B* in (1.7) can be obtained by selecting *M* appropriate rows of

$$\begin{aligned} B^{\mathrm {ext}}:= Q^{\mathrm {ext}}(\varLambda ^{\mathrm {ext}})^{1/2} . \end{aligned}$$

The use of FFT allows fast computation of the matrix-vector product $B^{\mathrm {ext}}{\varvec{y}}$ for any vector ${\varvec{y}}$, which then yields $B{\varvec{y}}$ needed for sampling the random field in (1.6). Our algorithm from [13] for obtaining a minimal positive definite $R^{\mathrm {ext}}$ is given in Algorithm 1. Our algorithm from [13] for sampling an instance of the lognormal random field is given in Algorithm 2. Note that the normalisation used within the FFT routine differs among particular implementations. Here, we assume the Fourier transform to be unitary.

#### Algorithm 1

Input: *d*, $m_0$, and covariance function $\rho$.

1. Set $$m = m_0$$.
2. Calculate ${\varvec{r}}$, the first column of $R^{\mathrm {ext}}$ in (3.20).
3. Calculate ${\varvec{v}}$, the vector of eigenvalues of $R^{\mathrm {ext}}$, by *d*-dimensional FFT on ${\varvec{r}}$.
4. If the smallest eigenvalue $<0$ then increment *m* and go to Step 2.

Output: *m*, $${\varvec{v}}$$.

#### Algorithm 2

Input: *d*, $m_0$, mean field $\overline{Z}$, and *m* and ${\varvec{v}}$ obtained by Algorithm 1.

1. With $s = (2m)^d$, sample an *s*-dimensional normal random vector ${\varvec{y}}$.
2. Update ${\varvec{y}}$ by elementwise multiplication with $\sqrt{{\varvec{v}}}$.
3. Set ${\varvec{w}}$ to be the *d*-dimensional FFT of ${\varvec{y}}$.
4. Update ${\varvec{w}}$ by adding its real and imaginary parts.
5. Obtain ${\varvec{z}}$ by extracting the appropriate $M=(m_0+1)^d$ entries of ${\varvec{w}}$.
6. Update ${\varvec{z}}$ by adding $\overline{Z}$.

Output: $$\exp ({\varvec{z}})$$.

In the case of QMC sampling, the random sample ${\varvec{y}}$ in Step 1 of Algorithm 2 is replaced by a randomly shifted lattice point from $[0,1]^s$, mapped to $\mathbb {R}^s$ elementwise by the inverse of the cumulative normal distribution function (see (1.12)). The relative size of the quantities $b_j = \Vert \mathbf {B}_j\Vert _\infty$ (as defined in (3.6)) determines the ordering of the QMC variables in order to benefit from the good properties of lattice rules in earlier coordinate directions in the construction of the generating vector in Remark 1.

We prove in [13] (under mild conditions) that Algorithm 1 will always terminate. Moreover, in many cases the required *m* (equivalently $\ell$) can be quite small. Theorem 7 below gives an explicit lower bound for the required value of $\ell$.

#### Example 1

The Matérn family of covariances are defined by

3.21 $$\begin{aligned} \rho ({\varvec{x}}) = \ \sigma ^2 \, \frac{2^{1-\nu }}{\varGamma (\nu )} \bigg (\frac{\sqrt{2\nu }}{\lambda }\, \Vert {\varvec{x}}\Vert _2\bigg )^{\nu } K_\nu \bigg ( \frac{\sqrt{2\nu }}{\lambda }\, \Vert {\varvec{x}}\Vert _2\bigg )\,, \end{aligned}$$

where $\varGamma$ is the Gamma function and $K_\nu$ is the modified Bessel function of the second kind, $\sigma ^2$ is the variance, $\lambda$ is the correlation length and $\nu \ge 1/2$ is a smoothness parameter. The limiting cases $\nu \rightarrow 1/2$ and $\nu \rightarrow \infty$ correspond to the exponential and Gaussian covariances respectively, see, e.g., [11], however, using a slightly different scaling.

The following result, proved in [13, Thm. 2.10], shows that the growth of the size of $\ell$ with respect to the mesh size $h_0$ and with respect to the parameters in the Matérn family is moderate. In particular, for fixed $\nu < \infty$, it establishes a bound on $\ell$ that grows only logarithmically with $\lambda /h_0$ and gets smaller as $\lambda$ decreases. Experiments illustrating the sharpness of this bound are given in [13].

#### Theorem 7

Consider the Matérn covariance family (3.21) with $1/2 \le \nu < \infty$ and $\lambda \le 1$. Suppose $h_0/\lambda \le e^{-1}$. Then there exist constants $C_1>0$ and $C_2\ge 2 \sqrt{2}$ which may depend on *s* but are independent of $\ell , h_0, \lambda , \nu$ and $\sigma ^2$, such that $R^{\mathrm {ext}}$ is positive definite if

$$\begin{aligned} \ell /\lambda \ \ge \ C_1\ + \ C_2\, \nu ^{1/2} \, \log \left( \max \{ {\lambda }/{h_0}, \, \nu ^{1/2}\} \right) \ . \end{aligned}$$

In the case $\nu =\infty$, the bound on $\ell$ is of the form $\ell \ge 1 + \lambda \, \max \{ \sqrt{2}\lambda /h_0, C_1\}$.

In order to verify the QMC convergence estimate given in Theorem 5 in the case of circulant embedding, we need to bound $\Vert {\varvec{b}}\Vert _{p,s}$, where ${\varvec{b}}$ is defined in (3.6). Since every entry in $\mathfrak {Re}(\mathcal {F}) + \mathfrak {Im}(\mathcal {F})$ is bounded by $\sqrt{2/s}$, we have

3.22 $$\begin{aligned} b_j \,=\, \Vert \mathbf {B}_j\Vert _\infty \,\le \, \sqrt{\frac{2}{s}\,\varLambda ^\mathrm{ext}_{s,j}}\,, \end{aligned}$$

where $\varLambda ^\mathrm{ext}_{s,j}$, $j=1,\ldots ,s$, are the eigenvalues of the nested block circulant matrix $R^{\mathrm {ext}}$. Notice that we added ‘*s*’ explicitly to the notation to stress the dependence of these eigenvalues on *s*. A sufficient condition to ensure the uniform boundedness of $\Vert {\varvec{b}}\Vert _{s,p}$ required in Theorem 5 is that there exists a constant $C>0$, independent of *s*, such that

3.23 $$\begin{aligned} \sum _{j=1}^{s} \left( \frac{\varLambda ^\mathrm{ext}_{s,j}}{s} \right) ^{p/2} \ \le \ C \ . \end{aligned}$$

It is thus important to investigate for what values of *p* this inequality holds. The smaller the value of *p* the faster the convergence will be in Theorem 5.

In [13, §3], we conjecture (with supporting mathematical arguments and empirical evidence) that the eigenvalues $\varLambda ^\mathrm{ext}_{s,j}$, when rearranged in non-increasing order, decay like $j^{-(1+2\nu /d)}$ in case of the Matérn covariance. This is the same as the decay rates of both the eigenvalues of the original nested block Toeplitz matrix *R* and of the KL eigenvalues of the underlying continuous field *Z*. Under this conjecture, it follows that the smallest value of *p* allowed for (3.23) to hold is just bigger than $2/(1+2\nu /d)$. In turn this yields a theoretical convergence rate of nearly

$$\begin{aligned} \mathcal {O}(n^{-\min (\nu /d,1)}) \end{aligned}$$

in Theorem 5 above, for any $\nu > d/2$, independently of *s*. To see this, recall that the convergence rate of $-1/(2\kappa )$ with respect to *n* in Theorem 5 is related to *p* via $p=2\kappa /(1+\kappa )$ with $\kappa \in (1/2,1)$. These bounds on $\kappa$ imply that, for the conjectured rate of decay of the eigenvalues $\varLambda ^\mathrm{ext}_{s,j}$, Theorem 5 is only applicable for $\nu > d/2$.

These conjectures will be investigated in detail in our numerical experiments in the next section. As we will see there, the theoretically predicted rates may be pessimistic. In the experiments here, we see nearly optimal QMC convergence, i.e., $\mathcal {O}(n^{-1})$, even when $\nu < d$, and at least as good convergence as for standard MC, i.e., $\mathcal {O}(n^{-1/2})$, even when $\nu < d/2$. All these findings are in line with the results we obtained in the case of KL expansions in [11], and they guarantee a dimension-independent optimal QMC convergence for sufficiently large smoothness parameter $\nu$.

## Numerical experiments

In this section we perform numerical experiments on problem (1.1) in 2D and 3D which illustrate the power of the proposed algorithm. Our quantity of interest will be the average value of the solution *u*

4.1 $$\begin{aligned} \mathcal {G}(u(\cdot ,{\varvec{y}})) \ = \ \frac{1}{\vert T \vert } \int _T u({\varvec{x}},{\varvec{y}}) \, \mathrm {d}{\varvec{x}}\ , \end{aligned}$$

over some measurable $T\subseteq D$, with *D* being an *L*-shaped domain with a hole in 2D or the unit cube in 3D; all details to be specified below. In both cases, the domain *D* is contained in the unit cube $[0,1]^d$, as assumed.

**Random field generation.** In all experiments the random coefficient *a* is of the form (1.2) where *Z* is a Gaussian random field with the Matérn covariance (3.21). We take the mean $\overline{Z}$ to be 0, the variance to be $\sigma ^2 = 0.25$, and we consider two different values for the correlation length, namely $\lambda \in \{0.2, 0.5\}$, combined with three different values for the smoothness parameter $\nu \in \{0.5, 2, 4\}$ in 2D and $\nu \in \{0.5, 3, 4\}$ in 3D (thus illustrating the cases $\nu <d$, $\nu =d$, $\nu >d$ in each case). The forcing term is taken to be $f\equiv 1$.

For different values of $m_0$, we first obtain values of the random field on a uniform grid with $(m_0+1)^d$ points on the unit cube $[0,1]^d$ by circulant embedding as described in Sect. 3.4. We choose $m_0\in \{12, 24, 48, 96\}$ in 2D and $m_0\in \{7, 14, 28\}$ in 3D. The necessary length $\ell = m/m_0$ of the extended cube $[0,\ell ]^d$ to ensure positive definiteness, where $m\ge m_0$, depends on the values of *d*, $\lambda$ and $\nu$, and affects the dimensionality $s=(2m)^d$ of ${\varvec{y}}$. This dependence is investigated in detail in [13] (see Theorem 7). In Table 1, we summarise the values of *s* for the different combinations of parameters.

**Table 1:** The values of *s* needed to ensure positive definiteness for different combinations of parameters in 2D and 3D

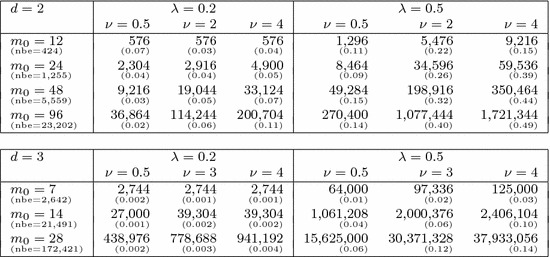	

The numbers in round brackets show the cost of random field generation as a fraction of the total computational cost per sample. These numbers increase with increasing *s* (from left to right) for a fixed FE mesh. For reference, we also provide the number of elements (nbe) for each of the FE meshes

**Fig. 1:**
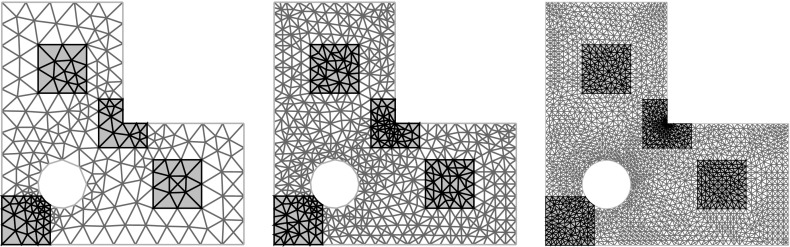
Adaptive FE mesh of an *L*-shaped domain with a hole. Left: $h=0.12$ (424 elements). Middle: $h=0.06$ (1255 elements). Right: $h=0.03$ (5559 elements). Our fourth FE mesh not shown here: $h = 0.015$ (23,202 elements)

**Fig. 2:**
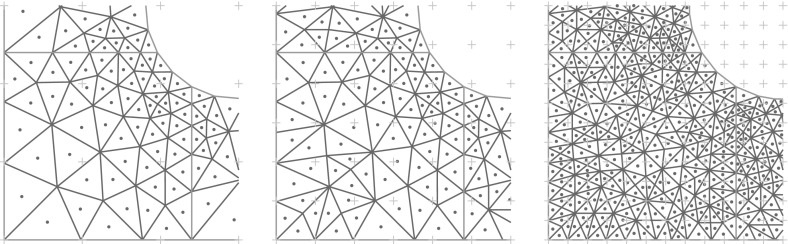
A local view of the meshes from Fig. 1, showing the quadrature points at the centroids of the triangles (blue dots) and the uniform grid points where the random field is sampled (purple crosses). Left: $(m_0,h)=(12,0.12)$. Middle: $(m_0,h)=(24,0.06)$. Right: $(m_0,h)=(48,0.03)$ (color figure online)

**FE solution of the PDE.** For each realisation of the random field (i.e., for each ${\varvec{y}}$), we solve the PDE using a finite element (FE) method with piecewise linear elements on an unstructured grid produced with the help of the Matlab PDE toolbox. The quadrature rule for the matrix assembly is based on the mid point rule, where the values of the random field at the centroids of the elements are obtained by multi-linear interpolation of the values on the uniform grid, computed with circulant embedding (see (2.6) and (2.7)). In order to balance the quadrature error and the FE discretisation error in light of Lemma 1 and Theorem 1, the maximum FE mesh diameter is chosen such that $h\approx \sqrt{d}\,h_0 = \sqrt{d}/m_0$. In particular, we choose $h\in \{0.12, 0.06, 0.03, 0.015\}$ in 2D and $h\in \{0.24, 0.12, 0.06\}$ in 3D, for each of the respective values of $m_0$ above. In 2D, the Matlab function adaptmesh is used to build a family of adaptive meshes for the *L*-shaped domain with a hole (see Fig. 1). We use the same adaptive mesh, constructed with $a \equiv 1$, for all realisations. To find meshes with our desired maximum mesh diameters *h*, we gradually increase the maxt parameter of the Matlab adaptmesh command. Figure 2 zooms in on the shaded region in the bottom left corner of each of the adaptive meshes to show the centroids of the triangles in relation to the uniform grids. The PDE is solved with the Matlab function assempde. For the 3D problem, we use the Matlab PDE toolbox to mesh and solve the PDE. The integral in (4.1) is approximated by applying the midpoint rule on each of the elements in *T*. In 2D, the resulting linear system is solved with the default sparse direct solver (“backslash”) in Matlab. We believe that that is also the solver used in the Matlab PDE toolbox for our 3D experiments, but we could not verify this.

As we can see from the fraction of time needed to construct the random field, which is shown in brackets in Table 1 for each case, the majority of time is spent on assembling and solving the FE systems. As expected this is even more pronounced in 3D, since sparse direct solvers are known to be significantly more expensive for 3D FE matrices with respect to the number of unknowns (both in terms of the order and in terms of the asymptotic constant), while the cost of the FFT factorisation grows log-linearly with the number of unknowns in 2D and in 3D. In any case, the random field generation is insignificant in the majority of cases and it takes less than 50% of the computational time in all cases.

**Construction of lattice sequences.** We approximate the expected value of (4.1) by randomly shifted lattice rules obtained using the fast CBC code from the QMC4PDE website https://people.cs.kuleuven.be/~dirk.nuyens/qmc4pde/ (see also [16]). A typical call to the QMC4PDE construction script would be:

./lat-cbc.py –s=2000 –b_file=[f] –m=16 –d1=1 –d2=2 –a3=1 –outputdir=[o]

where s=2000 specifies an initial maximum number of dimensions and [f] is a file containing the calculated values of $b_j$ in (3.6) in nonincreasing order for a particular case of *d*, $\lambda$ and $\nu$. See [16] for an explanation of the other parameters.

In specifying parameters for the CBC construction, we follow the theory of [16] as closely as possible, but we make a couple of modifications for practical reasons. Firstly, the implementation follows [8] to construct “embedded” lattice sequences in base 2, so that in practice we can increase *n* gradually without throwing away existing function evaluations. At every power of 2, the points form a full lattice rule which complies with the theory from Sect. 3. Secondly, with the POD weights in (3.16) the CBC construction to find the generating vector ${\varvec{z}}$ has a cost of $\mathcal {O}(s^2n + sn\log n)$ operations which becomes quite high for the large *s* we are considering (see Table 1). Thus, we only carry out the CBC construction up to a certain dimension $s^*$ and then randomly generate the remaining components of ${\varvec{z}}$. In particular, we stop the CBC algorithm at the first component $s^*$ where the generating vector has a repeated entry. Repeated components in ${\varvec{z}}$ yield bad two-dimensional projections of lattice points and randomly generated components are intuitively better in that situation. The highest dimensionality for the switch-over dimension for all cases in Table 1 is $s^*=1811$. The only two cases where we did not need to add random components were $\nu =2$ and $\nu =4$, for $d=2$, $m_0=12$ and $\lambda = 0.2$.

**Estimation of (Q)MC error.** For fixed *h*, we can compute the standard error on the QMC estimate of the expected value of (4.1) by using a number of random shifts. Specifically, for each case we took $q=64$ independent random shifts of one *n*-point lattice rule, giving *q* independent approximations $Q_1,\ldots ,Q_q$ to the expected value. We take their average $\overline{Q} = (Q_1+\cdots +Q_q)/q$ as our final approximation, and we estimate the standard error on $\overline{Q}$ by $\sqrt{\frac{1}{q(q-1)}\sum _{i=1}^q (Q_i-\overline{Q})^2}$. The total number of function evaluations in this case is $N = q\,n$. According to our theory (see also [16]), the convergence rate for our randomised QMC method is of the order $q^{-1/2}\,n^{-r} = q^{r-1/2}\,N^{-r}$, with $r\approx \min (\nu /d,1)$. Hence, for $r > 1/2$, the constant in any of the convergence graphs with respect to *N* depends on $q^{r-1/2}$. To provide less erratic curves, the number of random shifts is chosen to be fairly large here. In practice, e.g., $q=16$ shifts would be sufficient. This would effectively push all convergence graphs down, leading to bigger gains for QMC.

We compare QMC with the simple Monte Carlo (MC) method (1.14) based on *N* random samples. Denoting the function values for these samples by $Y_1,\ldots Y_N$, then the MC approximation of the integral is $\overline{Y} = (Y_1+\cdots + Y_N)/N$. The standard error can be estimated by $\sqrt{\frac{1}{N(N-1)} \sum _{i=1}^N (Y_i-\overline{Y})^2}$. The expected MC convergence rate is $\mathcal {O}(N^{-1/2})$.

In our figures later, we plot the *relative standard error* obtained by dividing the estimated standard error by the estimated mean.

**Computing environment.** All our computations were run as serial jobs on reserved 8-core Intel Xeon E5-2650v2 2.60 GHz nodes on the computational cluster Katana at UNSW (or on almost identical hardware). Since they are embarrassingly parallel, both the MC and QMC simulations could easily be parallelised with roughly linear speedup. We chose to run different jobs in parallel instead of parallelising individual jobs, and to report the actual serial computation times for our test cases.

### Results for an *L*-shaped domain in 2D

In this example the domain *D* is the complex 2D domain shown in Fig. 1: an *L*-shaped region with a hole. We consider five choices for the averaging domain $T \subseteq D$ in (4.1):

**T1**: the full domain,
**T2**: the bottom left corner with a circular segment cut out,
**T3**: the lower right interior square, and
**T4**: the upper left interior square in a symmetrical location to **T3**,
**T5**: the *L*-shape near the reentrant corner.

Figure 1 shows the different averaging domains *T*, as well as some of the adaptive meshes that were used. Note that the circular sections of the boundary of *D* are approximated polygonally and the averaging domains **T2**, ..., **T5** are resolved on all meshes. The meshes are adapted to capture the loss of regularity near the reentrant corner, but nevertheless the number of elements grows roughly with $\mathcal {O}(h^{-2})$, the same as for a uniform family of meshes with mesh size *h*. We specify the domain in Matlab by means of constructive solid geometry (CSG), i.e., the union and subtraction of the basic pieces. This ensures that all the averaging domains *T* are covered by complete elements.

**Mesh errors.** Before we compare the performance of our QMC method with the basic MC method, let us first estimate the discretisation errors for each of the adaptive meshes. In Table 2, we present results for **T1** and **T5** for the case $\lambda =0.2$ and $\nu =2$. The estimates of $\mathbb {E}[\mathcal {G}(u_h)]$, obtained using QMC, are stated together with the estimated standard error for each mesh. We use sufficiently many QMC cubature points, so that the significant figures of the estimates are not affected by QMC errors. The product of $m_0$ and *h* is kept fixed (approximately equal to $\sqrt{d}$), as discussed above. From the results we can clearly see that the convergence rate for the discretisation error is $\mathcal {O}(h^2)$ on the given meshes. This shows that the mesh refinement near the reentrant corner is working optimally. Using Richardson extrapolation, we can thus compute a higher order approximation of the limit of $\mathbb {E}[\mathcal {G}(u_h)]$ that is stated in the last row of Table 2. The relative error with respect to this extrapolated value is stated in the last column for both **T1** and **T5**.

**Table 2:** Mesh convergence for the case $\lambda =0.2$ and $\nu =2$ for **T1** and **T5**

	**T1**		**T5**	
$(m_0,h)$	$\mathbb {E}[\mathcal {G}(u_h)]$	rel. *h*-error	$\mathbb {E}[\mathcal {G}(u_h)]$	rel. *h*-error
(12, 0.12)	0.0114378 ± 3.4e−08	4.6e−02	0.0108212 ± 7.9e−08	7.9e−02
(24, 0.06)	0.0118095 ± 2.8e−08	1.5e−02	0.0114086 ± 6.6e−08	2.9e−02
(48, 0.03)	0.0119549 ± 4.1e−08	3.3e−03	0.0116850 ± 8.1e−08	5.9e−03
(96, 0.015)	0.0119846 ± 4.9e−08	8.3e−04	0.0117372 ± 1.1e−07	1.5e−03
	0.0119945 (extrapolated)	$\sim h^2$	0.0117546 (extrapolated)	$\sim h^2$

The estimates of $\mathbb {E}[\mathcal {G}(u_h)]$ (stated together with one standard deviation) are computed with our randomised lattice rule with *n* sufficiently large, such that the standard error is significantly smaller than the discretisation error. Richardson extrapolation is used to compute a more accurate estimate of the limit of $\mathbb {E}[\mathcal {G}(u_h)]$, as $h\rightarrow 0$ (final row). The columns denoted “rel. *h*-error” give the relative error with respect to these extrapolated estimates

The behaviour is similar for the other quantities of interest and for the other values of $\lambda$ when $\nu =2$. For $\nu =0.5$, on the other hand, the solution is globally only in $H^{3/2}$, so that the local mesh refinement near the reentrant corner plays no role and the convergence of $\mathbb {E}[\mathcal {G}(u_h)]$ is only $\mathcal {O}(h)$. Thus, to achieve acceptable accuracy, comparable to the QMC errors we quote below, we would in practice need much finer meshes for $\nu =0.5$. However, since this would not affect the behaviour of the QMC cubature errors, we did not do that.

**Fig. 3:**
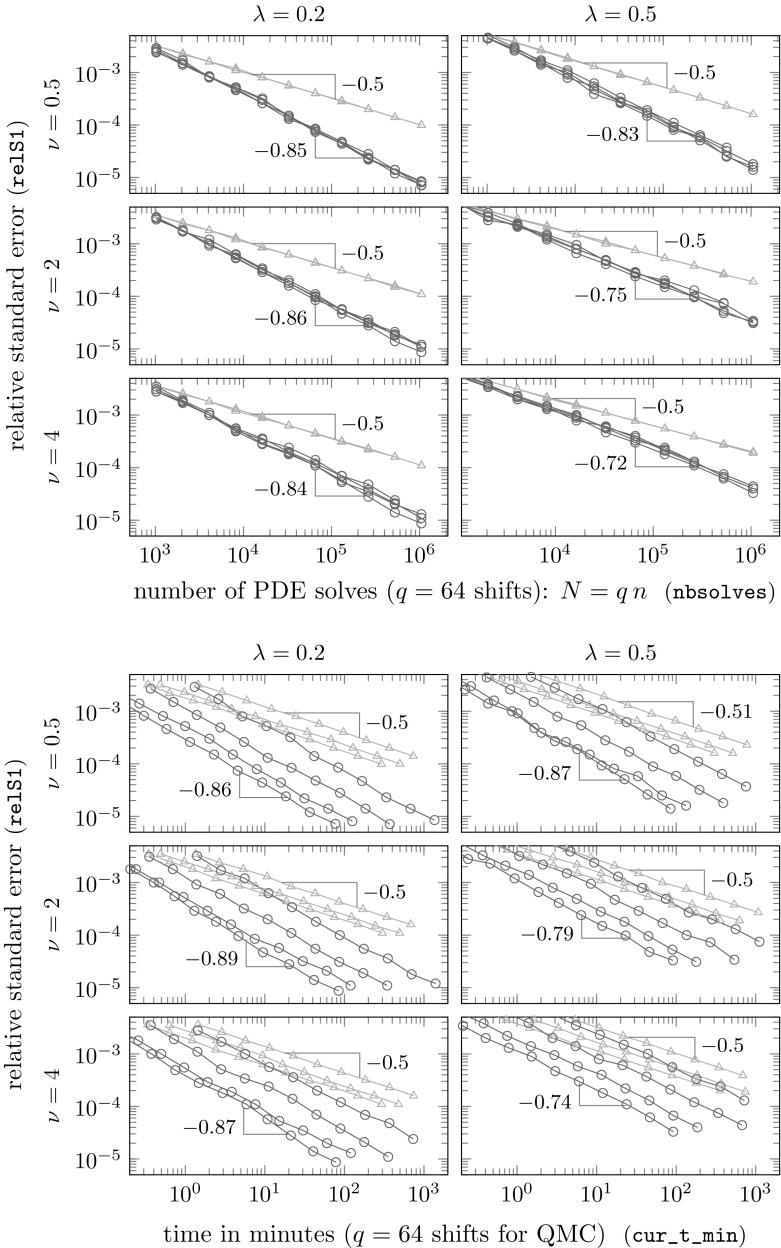
Relative standard error for $T=$ **T1** (the average solution over the entire domain) against total number of PDE solves (top) and execution time (bottom) for Monte Carlo (red triangles) and QMC (blue circles). In the timing plot, results appear from bottom to top from the coarsest mesh with $(m_0,h)=(12,0.12)$ to the inest mesh with $(m_0,h)=(96,0.015)$ for QMC and $(m_0,h)=(48,0.03)$ for MC, respectively (color figure online)

**Fig. 4:**
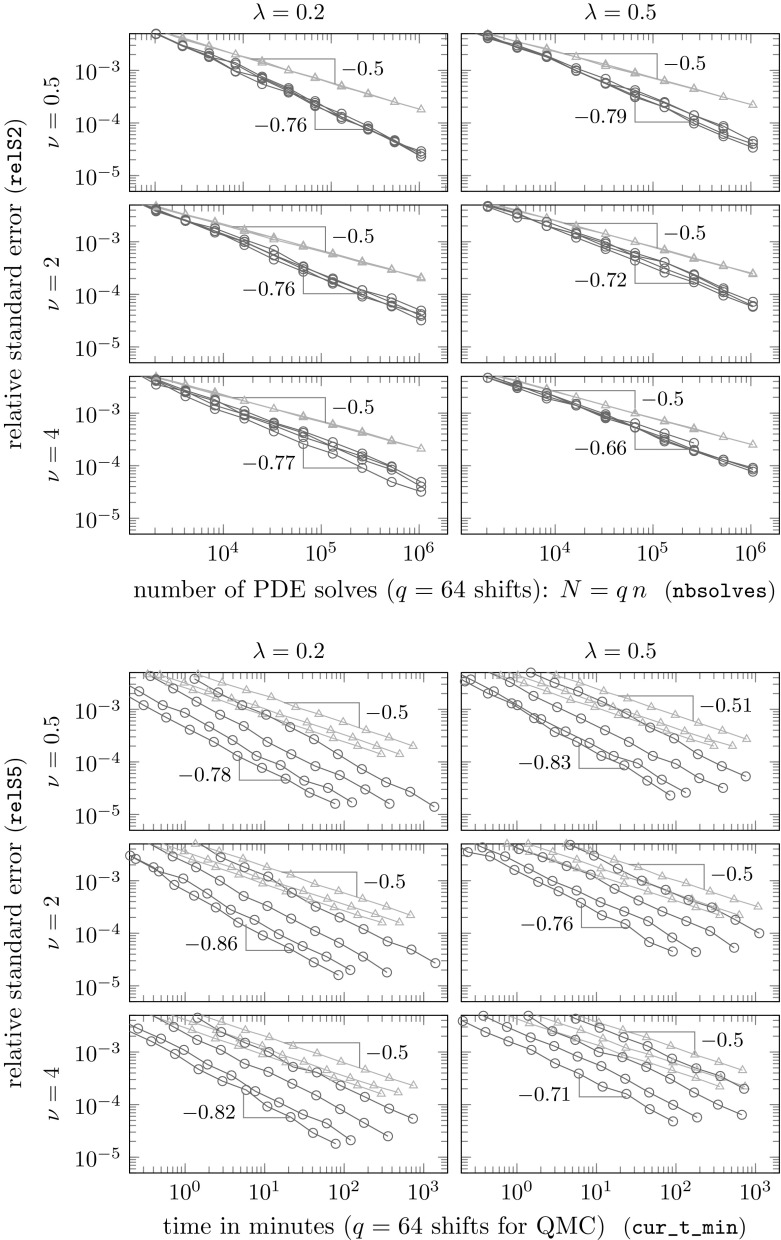
Relative standard error for Monte Carlo (red triangles) and the lattice sequences (blue circles) for **T2** (the average over the bottom-left corner with circular cutout) versus number of PDE solves (top), as well as for **T5** (the average over the L-shaped region near the reentrant corner) versus execution time (bottom). In the bottom figure, the results appear from bottom to top from the coarsest mesh to the finest (color figure online)

**Fig. 5:**
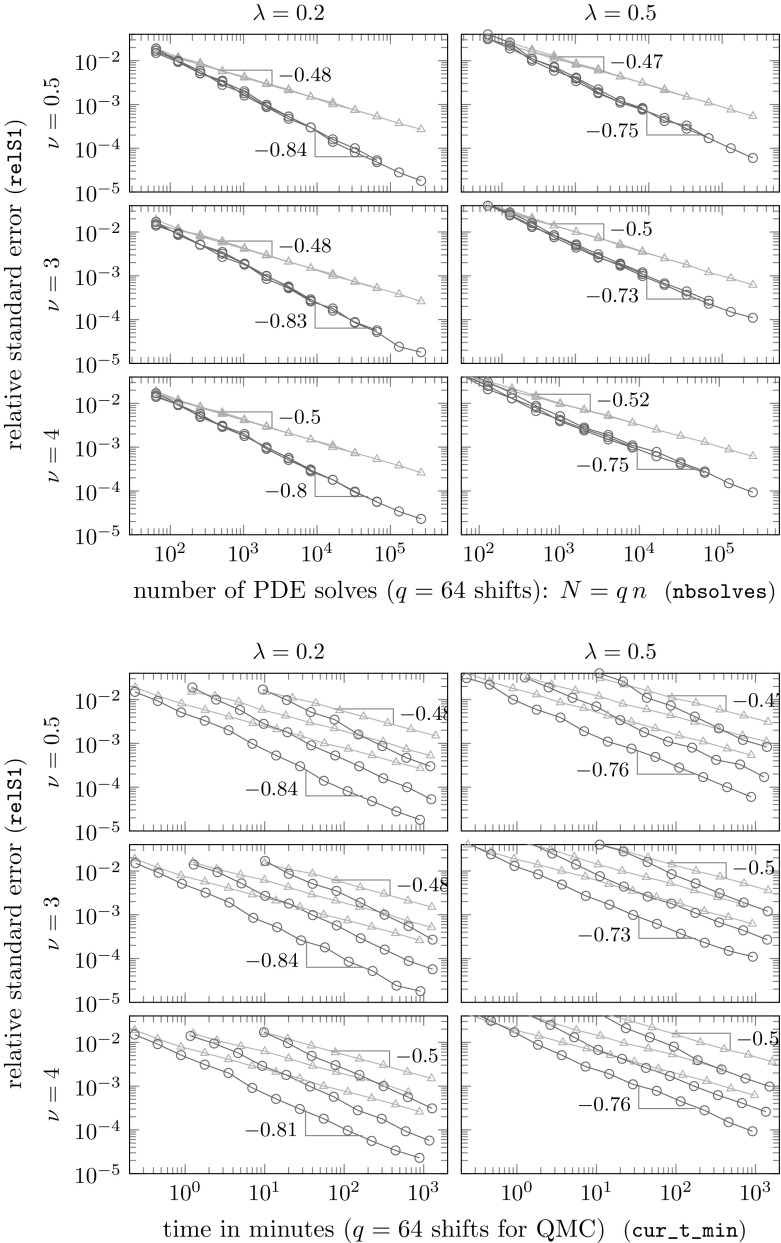
Relative standard error of the average solution over the unit cube in 3D against the total number of PDE solves (top) and the execution time (bottom) for Monte Carlo (red triangles) and the lattice sequences (blue circles). As in Fig. 3, the results appear from bottom to top from the coarsest mesh with $(m_0,h)=(7,0.24)$ to the finest mesh with $(m_0,h)=(28,0.06)$ (color figure online)

**QMC convergence rates.** For the remainder we will now demonstrate the dimension independence of our QMC method and its superiority over basic MC. In Fig. 3, we plot the relative standard error in (4.1) with $T=$ **T1** against the total number of PDE solves (top) and against the total calculation time (bottom). We consider six combinations of the values of $\nu$ and $\lambda$ and plot the graphs for four levels of mesh refinement. (The MC estimates were not computed on the finest mesh.) The convergence of the MC method is proportional to $N^{-0.5}$, as expected. The convergence of the QMC method ranges from $\mathcal {O}(N^{-0.72})$ up to $\mathcal {O}(N^{-0.89})$. For example, for $\nu =0.5$ and $\lambda =0.2$, to achieve the same relative standard error of $10^{-4}$ we need about $10^6$ PDE solves with the MC method while the QMC method only needs about $3\cdot 10^4$ PDE solves. Also in terms of computational time, all the results consistently show huge computational savings for the QMC method over the MC method, even with the relatively large number of $q=64$ random shifts.

We note that the convergence graphs are meant to illustrate the convergence behaviour and in practice one would not try to achieve such high precision, especially not on the coarser meshes. One would rather aim to balance the QMC errors with the discretisation errors in Table 2. From the theory, we expect the smoothness $\nu$ of the random field to have an effect on the convergence rate of QMC. Specifically, the bound in Theorem 5 would suggest a rate of $\mathcal {O}(N^{-\min (\nu /2,1)})$ in 2D, as discussed in Sect. 3.4. This effect is not immediately observed in the graphs. For $\nu =2$ and 4 we expect an asymptotic convergence rate of order 1, while we see very good convergence rates on the graphs, we did not reach this asymptotic regime yet. On the other hand, we do observe excellent convergence behaviour for the case $\nu =0.5$ for which our theory does not apply. From the graphs we also observe that a smaller correlation length $\lambda$ corresponds to a better convergence rate, which is in full agreement with our findings in [12].

A more important observation is the overlay of the convergence lines for the different meshes in the plots of relative standard error versus number of PDE solves in Fig. 3. For example, for the case $\lambda =0.5$ and $\nu =4$ the dimensionality *s* increases from about 9 thousand to 1 million as we increase $m_0$ from 12 to 96 (see Table 1), while the convergence rate and the asymptotic constant for the relative standard error are clearly independent of the increasing dimension.

In Fig. 4, we confirm that the superiority is independent of the quantity of interest, by presenting similar graphs as for **T1** also for **T2** and **T5**. We do not include the results for the two symmetrical squares **T3** and **T4**, which look very similar.

### Results for the unit cube in 3D

Our second example is the unit cube in 3D and our quantity of interest is (4.1) with $T = D = [0,1]^3$. We use the mesh generator and the solver from the Matlab PDE toolbox to obtain three meshes with desired maximum mesh diameters *h* and to calculate the solution. To evaluate the random field *Z* at the centroids of the elements we used the function interpolateSolution from the toolbox.

In Fig. 5 we plot again the relative standard error for six combinations of parameters against the total number of PDE solves and against the calculation time. We observe convergence rates from $N^{-0.73}$ up to $N^{-0.84}$ for our QMC rules, and the expected $N^{-0.5}$ for the plain MC method. The lattice sequences were again constructed with the actual values of $b_j$ from (3.6), except for the combination of $\lambda =0.5$ and $m_0 = 28$ where we get the near astronomical dimensionalities ranging from about 15 to 37 million, as $\nu$ increases (see Table 1); for these cases we replaced $b_j$ by the convenient upper bound given in (3.22).
